# Global *N*-linked Glycosylation is Not Significantly Impaired in Myoblasts in Congenital Myasthenic Syndromes Caused by Defective Glutamine-Fructose-6-Phosphate Transaminase 1 (GFPT1)

**DOI:** 10.3390/biom5042758

**Published:** 2015-10-16

**Authors:** Qiushi Chen, Juliane S. Müller, Poh-Choo Pang, Steve H. Laval, Stuart M. Haslam, Hanns Lochmüller, Anne Dell

**Affiliations:** 1 Department of Life Sciences, Faculty of Natural Sciences, Imperial College London, South Kensington Campus, London SW7 2AZ, UK; E-Mails: q.chen11@imperial.ac.uk (Q.C.); p.pang05@imperial.ac.uk (P.-C.P.); s.haslam@imperial.ac.uk (S.M.H.); 2 John Walton Muscular Dystrophy Research Centre, Institute of Genetic Medicine, Newcastle University, Newcastle Upon Tyne NE1 3BZ, UK; E-Mails: juliane.mueller@ncl.ac.uk (J.S.M.); shlaval@outlook.com (S.H.L.); Hanns.Lochmuller@newcastle.ac.uk (H.L.)

**Keywords:** congenital myasthenic syndromes, glutamine-fructose-6-phosphate transaminase 1, glycosylation, mass spectrometry

## Abstract

Glutamine-fructose-6-phosphate transaminase 1 (GFPT1) is the first enzyme of the hexosamine biosynthetic pathway. It transfers an amino group from glutamine to fructose-6-phosphate to yield glucosamine-6-phosphate, thus providing the precursor for uridine diphosphate *N*-acetylglucosamine (UDP-GlcNAc) synthesis. UDP-GlcNAc is an essential substrate for all mammalian glycosylation biosynthetic pathways and *N*-glycan branching is especially sensitive to alterations in the concentration of this sugar nucleotide. It has been reported that *GFPT1* mutations lead to a distinct sub-class of congenital myasthenic syndromes (CMS) termed “limb-girdle CMS with tubular aggregates”. CMS are hereditary neuromuscular transmission disorders in which neuromuscular junctions are impaired. To investigate whether alterations in protein glycosylation at the neuromuscular junction might be involved in this impairment, we have employed mass spectrometric strategies to study the *N*-glycomes of myoblasts and myotubes derived from two healthy controls, three *GFPT1* patients, and four patients with other muscular diseases, namely CMS caused by mutations in *DOK7*, myopathy caused by mutations in *MTND5*, limb girdle muscular dystrophy type 2A (LGMD2A), and Pompe disease. A comparison of the relative abundances of bi-, tri-, and tetra-antennary *N*-glycans in each of the cell preparations revealed that all samples exhibited broadly similar levels of branching. Moreover, although some differences were observed in the relative abundances of some of the *N*-glycan constituents, these variations were modest and were not confined to the GFPT1 samples. Therefore, *GFPT1* mutations in CMS patients do not appear to compromise global *N*-glycosylation in muscle cells.

## 1. Introduction

Protein glycosylation, the attachment of carbohydrate chains to proteins, is a common post-translational modification occurring ubiquitously in eukaryotic cells. Intriguingly, mutations in genes encoding protein glycosylation enzymes, or enzymes synthesising the building blocks for protein glycosylation, have recently been identified to cause neuromuscular transmission defects called congenital myasthenic syndromes (CMS). The first of these genes to be correlated with CMS was *GFPT1* (glutamine-fructose-6-phosphate transaminase 1). Mutations in *GFPT1* cause a distinct sub-class of CMS referred to as “limb-girdle CMS with tubular aggregates” [1,2]. Subsequently, mutations in three genes encoding enzymes of the protein *N*-glycosylation pathway (*DPAGT1*, *ALG2* and *ALG14*) were also found to cause limb-girdle CMS [3,4]. The *GFPT1* gene encodes a homodimeric, cytoplasmic enzyme that catalyses the first step of the hexosamine biosynthetic pathway [1]. Thus GFPT1 converts fructose-6-phosphate and glutamine into glucosamine-6-phosphate and glutamate; the end product of this pathway is uridine diphospho-*N*-acetylglucosamine (UDP-GlcNAc), which is a basic substrate not only for protein *N*- and *O*-linked glycosylation, but also for lipid glycosylation, proteoglycan synthesis, and O-GlcNAc glycosylation [1]. Currently it is not clear which of these pathways is affected most by lower levels of UDP-GlcNAc caused by GFPT1 deficiency. However, clinical presentation of CMS patients with mutations in *DPAGT1*, *ALG2* and *ALG14* is very similar to *GFPT1* CMS, suggesting that impaired protein *N*-glycosylation might be the molecular defect underlying *GFPT1* CMS. *N*-glycosylation is crucial for the assembly and function of a number of key molecules at the neuromuscular junction, like the acetylcholine receptor, the muscle-specific kinase (MuSK), and agrin. However, it is currently not clear why muscles, or more specifically the neuromuscular junctions, are more vulnerable than other tissues to GFPT1 deficiency.

We set out to determine whether protein *N*-glycosylation is impaired or modified in the muscles of CMS patients with *GFPT1* mutations. Specifically we employed mass spectrometric glycomic methodologies to rigorously characterise *N*-glycosylation in primary myoblasts from patients with *GFPT1* mutations, as well as myotubes obtained by their *in vitro* differentiation. As controls for these analyses, we analysed myoblasts from two healthy controls and four patients with muscular diseases that have not previously been linked to glycosylation, namely CMS caused by mutations in *DOK7*, myopathy caused by mutations in *MTND5*, limb girdle muscular dystrophy type 2A (LGMD2A) caused by mutations in the *CAPN3* gene encoding calpain-3, and Pompe disease caused by mutations in the *GAA* gene encoding acid maltase responsible for breaking down glycogen in lysosomes. Unexpectedly, no impairment of *N*-glycosylation was observed in the *GFPT1* patients’ myoblasts or myotubes.

## 2. Results and Discussion

### 2.1. Optimisation of Myoblast Culture Conditions

Due to the fact that muscle biopsies do not provide sufficient sample for glycomic analysis we first established suitable cell culture conditions for producing the required cell counts (>10^6^) whilst minimising the amount of foetal calf serum (FCS) present in the culture medium. The latter was important because it is known that FCS derived glycans frequently co-purify with cell-derived glycans during glycomic analyses [5]. We found that culturing in media containing 15% FCS was optimal for our glycomics experiments (see Figure S1).

### 2.2. Glycomic Analysis of Patient and Control Myoblasts Reveals no Impairment in N-Glycosylation

**Figure 1:**
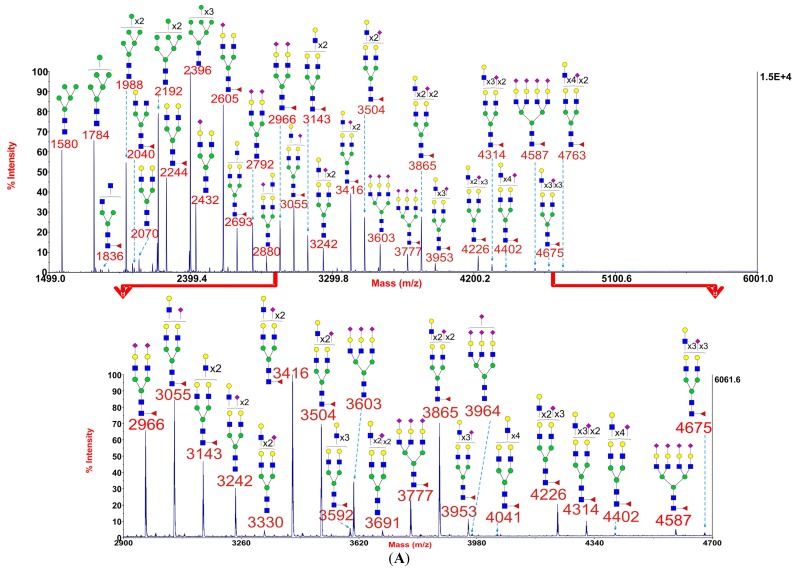

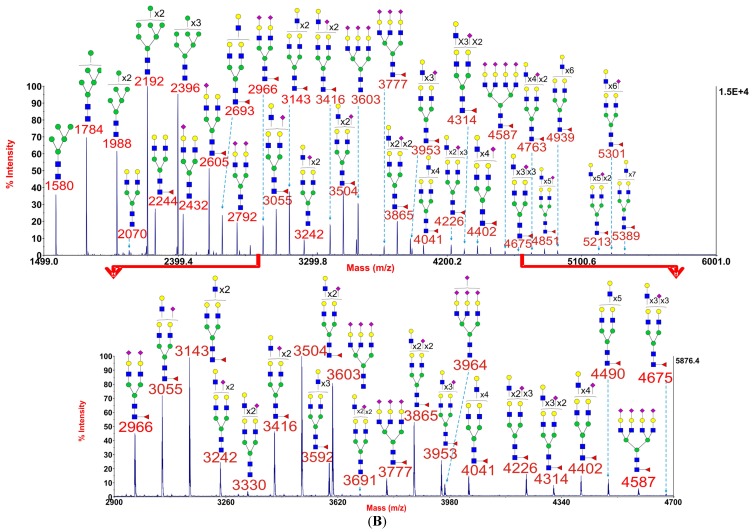

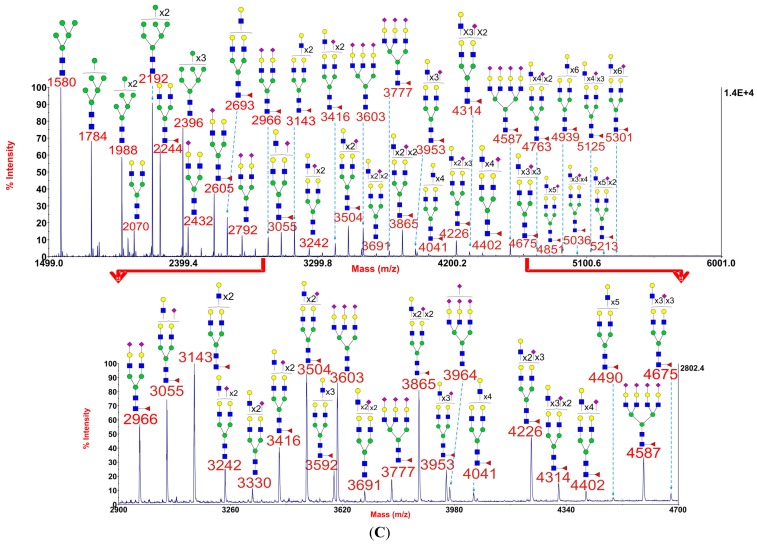
Annotated MALDI-TOF MS spectra of permethylated *N*-glycans of myoblasts from healthy control 1 (**A**), *GFPT1* patient 1 (**B**), and the *DOK7* patient (**C**). In each of A, B and C, the top panel shows the full spectrum of glycans and the bottom panel amplifies the mass range where the majority of tri- and tetra-antennary glycans are found, the starting point and ending point of which have been indicated by red arrows. Profiles were obtained from the 50% acetonitrile fraction from a C18 Sep-Pak column. All ions are [M + Na]^+^. The number indicated in the spectra is the mass to charge ratio (m/z) of the corresponding glycan ion. Since the ion is monocharged, the value of m/z is equal to the molecular weight value of the glycan. Annotations are based on the molecular weight, *N*-glycan biosynthetic pathway, and MS/MS data. Glycans at m/z 2966, 3777, and 4587 are clearly annotated, which is due to the fact that their structures are unequivocal because each antenna is capped with a sialic acid and thus they are homogeneous bi-, tri-, and tetraantennary glycans. However, the glycan structure is not always as unequivocal as the glycan at m/z 2966 as biosynthetically non-fully sialylated glycan molecular ion species could be made up of mixtures of structural isoforms. Therefore, for those heterogeneous multiantennary structures with extended LacNAc repeats, the annotations are simplified throughout by using biantennary structures with the extensions and NeuAcs listed outside a bracket. 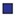 GlcNAc, 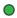 Man, 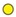 Gal, 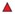 Fuc, 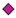 NeuAc.

Myoblasts were cultured from three *GFPT1* patients, one *DOK7* patient, one *MTND5* patient, one LGMD2A patient, one Pompe disease patient, and two healthy controls. MALDI-TOF *N*-glycomic profiling was performed on duplicate myoblast preparations with high quality data being acquired in all instances although minor glycans were not observed in *GFPT1* patient 3 whose myoblasts were difficult to culture. Representative MALDI-TOF spectra from a healthy control, a *GFPT1* patient and the *DOK7* patient are shown in Figure 1. MALDI data for the other patients and control are reproduced in Supplementary Information (see Figure S2).

Figure 1 shows that myoblast *N*-glycans comprise both high mannose and complex (bi-, tri-, and tetraantennary) structures. Some common characteristics of mammalian cell *N*-glycomes [6,7] were observed, such as core fucosylated GlcNAc, LacNAc antenna building blocks which, in some cases, are tandemly repeated to produce oligo-LacNAc extensions, and NeuAc-capped antennae. Significantly, the *N*-glycan profile of healthy control 1 showed a broadly similar pattern to those of *GFPT1* patient 1 and the *DOK7* patient.

**Figure 2:**
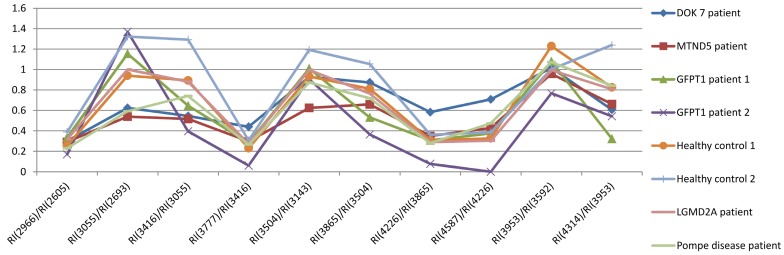
Comparison of *N*-glycan sialylation in the myoblasts. Each point in the graph indicates a ratio which was obtained by comparing the relative intensity (RI) of one glycan to that of another glycan which possesses one fewer NeuAc. The numbers in the brackets correspond to the m/z of the comparing glycans.

To investigate whether the *GFPT1* patients exhibited impaired *N*-glycan branching, we categorised the *N*-glycans into families with differing levels of sialylation and then compared glycan abundances within these families. We first checked that sialylation levels were similar in the different myoblast samples. We did this by calculating abundance ratios of pairs of glycans where one member of the pair had one more sialic acid than the other but otherwise the pair had identical compositions. The results of these calculations are shown in Figure 2 for the various myoblast samples. The data show that sialylation patterns are broadly similar for all samples.

**Figure 3:**
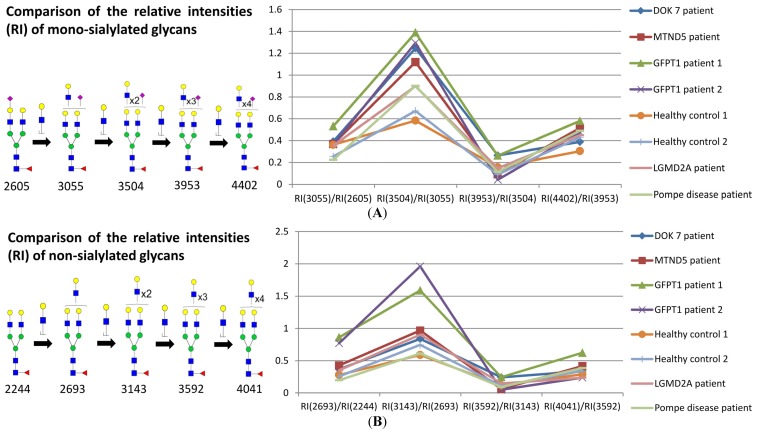
Comparison of the relative intensities of a family of mono-sialylated glycans with different numbers of LacNAc (**A**) in myoblasts; comparison of the relative intensities of a family of non-sialylated glycans with different numbers of LacNAc (**B**) in myoblasts. Each point in the graph indicates a ratio which was obtained by comparing the relative intensity (RI) of one glycan to that of the corresponding glycan which possesses one fewer LacNAc moiety. The number under the glycan structure is the m/z value of the glycan; this number is increasing with the addition of LacNAc moiety. In each comparison, the numbers in the brackets correspond to the m/z of the comparing glycans. 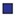 GlcNAc, 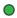 Man, 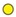 Gal, 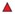 Fuc, 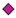 NeuAc.

For each family of sialylated glycans we then compared the abundance ratios of pairs of glycans differing in composition by a single LacNAc moiety. For example Figure 3 shows comparative data for mono-sialylated (panel A) and non-sialylated (panel B) glycans containing from two to six LacNAc units. All samples showed a similar profile of relative abundances, albeit there is a two to three-fold divergence of ratios when comparing four LacNAcs with three LacNAcs (for example m/z 3504 and 3055, panel A), with the GFPT1 samples showing somewhat higher levels of the former than observed in the other samples.

**Figure 4:**
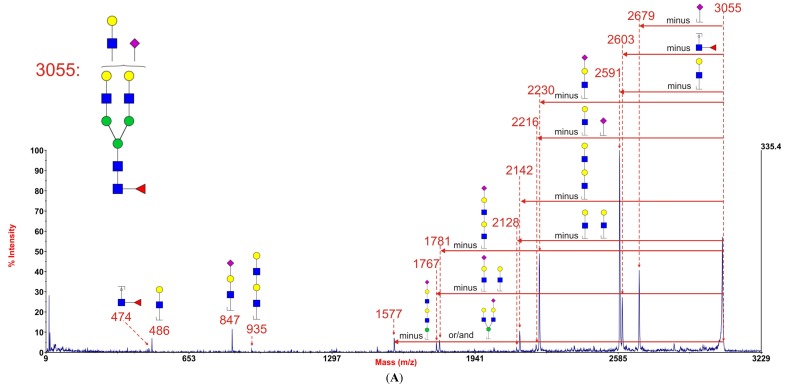

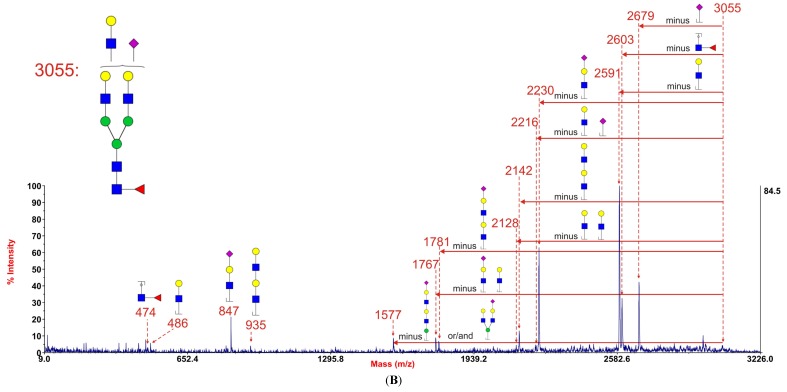

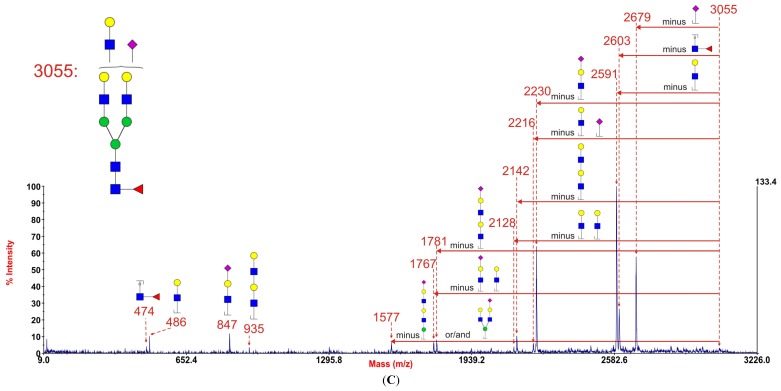
MALDI-TOF/TOF MS/MS spectra of the permethylated *N*-glycan at m/z 3055 [M + Na]^+^ in myoblasts from healthy control 1 (**A**), *GFPT1* patient 1 (**B**), and the *DOK7* patient (**C**). Assignments of the fragment ions are indicated on the cartoons and on the spectra the horizontal red arrows show antennae losses whilst antennae-derived fragment ions are annotated with their sequences. The number indicated above the peak in the spectra is the m/z value of the fragment ion (resulting ion) that has been detected by the mass spectrometry. 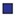 GlcNAc, 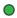 Man, 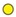 Gal, 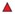 Fuc, 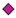 NeuAc.

These unexpected results did not support our hypothesis that branching might be impaired in *GFPT1* myoblasts. Indeed they suggested that multiantennary glycans were possibly slightly more abundant in *GFPT1* patients than in controls. However, it is important to note that increasing numbers of LacNAc units is not necessarily indicative of increased branching because these moieties can be present in extended oligo-LacNAc antennae rather than as additional antennae. Indeed the MALDI data in Figure 1 confirm that myoblasts are capable of extending their antennae because many of the glycans at high mass have more than the four LacNAc moieties that are the basis of a tetra-antennary glycan. Fortunately isomeric glycans differing in branching and oligo-LacNAc extensions can be readily distinguished and their abundances compared by analysing characteristic fragment ions in MS/MS experiments. For example Figure 4 shows MS/MS spectra obtained from the monosialylated glycan with three lacNAc units (m/z 3055) in the MALDI data (see Figure 1) for healthy control 1, *GFPT1* patient 1 and the *DOK7* patient. Two antennae arrangements are consistent with this composition: triantennary and/or biantennary with one LacNAc extension. As shown in the annotations on the spectra and the cartoons in Figure 4, the MS/MS spectra are dominated by fragment ions arising from loss of a single terminal LacNAc, with or without sialic acid. These fragment ions can be derived from both the bi-and tri-antennary options. Nevertheless, there are several fragment ions that are diagnostic for the extended biantennary structure. These are observed at m/z 935, 1781, and 2142. All are minor. Importantly, their abundances relative to the major fragment ions are similar in the three samples. We estimate from abundance comparisons that m/z 3055 is comprised of about 90% tri-antennary and 10% extended bi-antennary structures in all cases.

MS/MS analyses of other glycans with similarly ambiguous compositions gave results comparable to those described above (see Figure S3). Taken together, the MS and MS/MS data provide convincing evidence that the patterns of branching and/or antennae extensions are very similar amongst the myoblasts from *GFPT1* patients, healthy controls, and the other muscle disease patients studied.

### 2.3. Sialic Acid Linkages Are Predominantly α2-3 in Myoblasts

**Figure 5:**
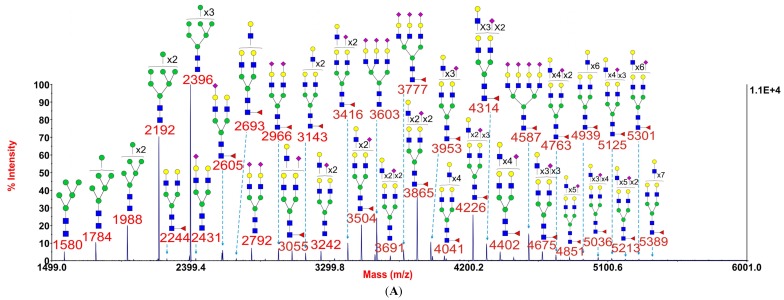

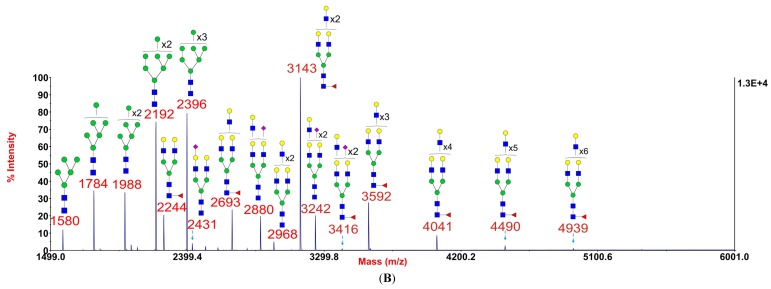
Annotated MALDI-TOF MS spectra of permethylated *N*-glycans (**A**) and sialidase S treated *N*-glycans (**B**) of myoblasts from the *DOK7* patient. In each of A, B and C, the top panel shows the full spectrum of glycans and the bottom panel amplifies the mass range where the majority of tri- and tetra-antennary glycans are found, the starting point and ending point of which have been indicated by red arrows. Profiles were obtained from the 50% acetonitrile fraction from a C18 Sep-Pak column. All ions are [M + Na]^+^. The number indicated in the spectra is the mass to charge ratio (m/z) of the corresponding ion. Since the ion is monocharged, the value of m/z is equal to the molecular weight value of the glycan. Annotations are based on the molecular weight, *N*-glycan biosynthetic pathway and MS/MS data. Glycans at m/z 2966, 3777, and 4587 are clearly annotated, which is due to the fact that their structures are unequivocal because each antenna is capped with a sialic acid and thus they are homogeneous bi-, tri-, and tetraantennary glycans. However, the glycan structure is not always as unequivocal as the glycan at m/z 2966 as biosynthetically non-fully sialylated glycan molecular ion species could be made up of mixtures of structural isoforms. Therefore, for those heterogeneous multiantennary structures with extended LacNAc repeats, the annotations are simplified throughout by using biantennary structures with the extensions and NeuAcs listed outside a bracket. 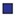 GlcNAc, 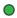 Man, 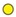 Gal, 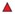 Fuc, 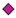 NeuAc.

To obtain information on sialic acid linkages we carried out sialidase S digestion on a myoblast preparation from the *DOK7* patient. This sialidase is specific for α2-3 linked sialic acid. As shown in Figure 5, digestion with sialidase S resulted in nearly complete desialylation of all of the core fucosylated *N*-glycans. A handful of minor sialylated glycans were observed at m/z 2431, 2880, and 3242. These α2-6 linked sialylated bi- and tri-antennary glycans are lacking core fucose and are likely to be derived from FCS in the culture medium (see Section 2.1). We conclude that the sialic acid in the myoblast *N*-glycans is mainly α2-3 linked.

### 2.4. Glycomic Analysis of Patient and Control Myotubes Reveals no Significant Difference in N-glycosylation

**Figure 6:**
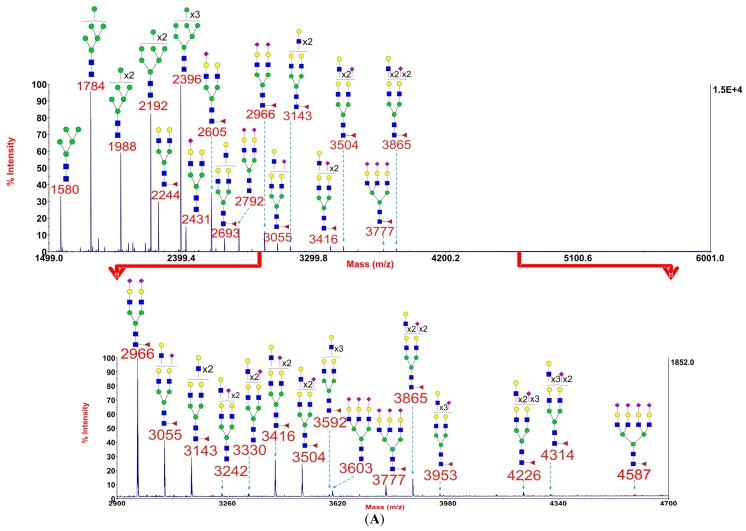

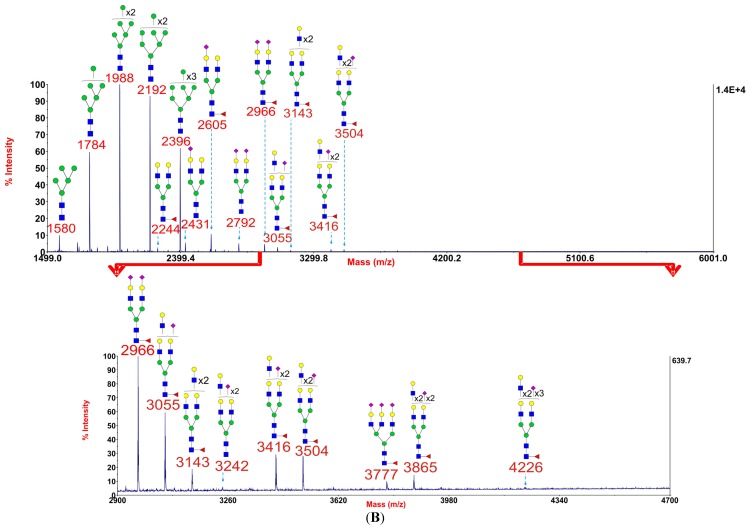

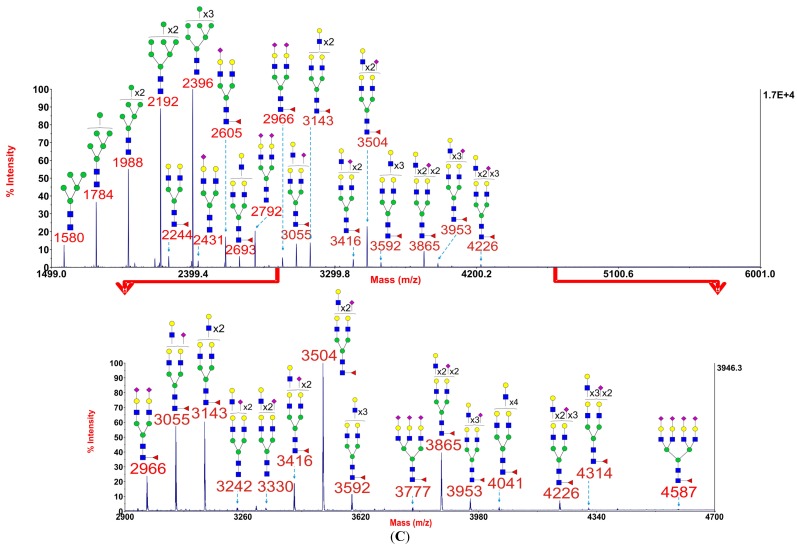
Annotated MALDI-TOF MS spectra of permethylated *N*-glycans of myotubes from healthy control 1 (**A**); *GFPT1* patient 1 (**B**) and the *DOK7* patient (**C**). In each of A, B and C, the top panel shows the full spectrum of glycans and the bottom panel amplifies the mass range where the majority of tri- and tetra-antennary glycans are found, the starting point and ending point of which have been indicated by red arrows. Profiles were obtained from the 50% acetonitrile fraction from a C18 Sep-Pak column. All ions are [M + Na]^+^. The number indicated in the spectra is the mass to charge ratio (m/z) of the corresponding ion. Since the ion is monocharged, the value of m/z is equal to the molecular weight value of the glycan. Annotations are based on the molecular weight, *N*-glycan biosynthetic pathway and MS/MS data. Glycans at m/z 2966, 3777 and 4587 are clearly annotated, which is due to the fact that their structures are unequivocal because each antenna is capped with a sialic acid and thus they are homogeneous bi-, tri- and tetraantennary glycans. However, the glycan structure is not always as unequivocal as the glycan at m/z 2966 as biosynthetically non-fully sialylated glycan molecular ion species could be made up of mixtures of structural isoforms. Therefore, for those heterogeneous multiantennary structures with extended LacNAc repeats, the annotations are simplified throughout by using biantennary structures with the extensions and NeuAcs listed outside a bracket. 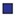 GlcNAc, 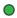 Man, 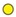 Gal, 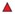 Fuc, 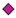 NeuAc.

Myotubes were obtained from all patients and controls by *in vitro* differentiation of their myoblasts in culture. They were then subjected to the same glycomic analyses as for myoblasts. Representative MALDI spectra are shown for healthy control 1, *GFPT1* patient 1, and the *DOK7* patient in Figure 6; spectra from other samples can be found in Supplementary Information (see Figure S4). Most samples gave good quality MALDI data, but, because sample quantities were more limited than for the myoblast preparations, low abundance glycans at high mass were not always observed. Nonetheless, we were able to draw firm conclusions from our data. Notably no significant differences were observed in the MALDI profiles from normal and GFPT1 samples (Figure 6A,B). Both showed a predominance of biantennary glycans, relatively high levels of tri- and tetraantennary glycans, and only very minor signals for glycans with more than four lacNAc units. The DOK7 myotubes similarly showed only minor signals for extended glycans but, in contrast to the normal and GFPT1 myotubes, their tri- and tetraantennary glycans were more abundant than biantennary structures. Similar glycomic profiling was performed on cells from healthy controls, GFPT1, MTND5, and LGMD2A patients. They all showed broadly similar glycan profiles as those observed in Figure 6A,B (see Figure S4).

### 2.5. Discussion

This is the first report of *N*-glycan profiles of *in vitro* cultured human myoblasts and human myotubes differentiating from myoblasts *in vitro*. The main findings of this work are that the *N*-glycan profiles of myoblasts (Figure 1) and myotubes (Figure 6) from *GFPT1* patients and a selection of healthy and disease controls are broadly similar. All have comparable levels of high mannose and complex-type structures. The latter are core fucosylated and capped with α2-3 linked sialic acid. The relative abundances of the bi-, tri- and tetraantennary complex-type glycans are broadly similar in all samples except for the DOK7 myotubes whose multiantennary glycans were found to be somewhat more abundant than the other samples (Figure 6).

These findings are unexpected as our initial hypothesis was that cells from *GFPT1* patients would have changes in their *N*-glycan structures and particularly a reduction in *N*-glycan branching. This was based on data we recently published in which we characterised *N*-glycosylation of leukocytes from patients with mutations in phosphoglucomutase 3 (*PGM3*), another key enzyme in the biosynthesis of UDP-GlcNAc. A reduction in levels of tri-antennary and tetra-antennary *N*-glycans was observed which was rationalized by the fact that if a comparison is made of the GlcNAc transferases that are responsible for *N*-glycan branching the higher Km values (which in Michaelis-Menten kinetics corresponds to low substrate affinity and thus a low catalytic activity), are associated with GlcNAc transferase IV and GlcNAc transferase V that initiate the third and fourth antennae. Therefore by requiring higher substrate concentrations they will be most impaired by a reduction in UDP-GlcNAc concentration [8].

Our findings that glycomic profiling of cells from *GFPT1* patients is not altered does not however rule out that changes in *N*-glycosylation still plays role in the associated disease pathologies. It could be that occupancy of *N*-glycosylation sites is altered in the patients. The glycomic methods employed in the current work do not address occupancy. Indeed, assigning *N*-glycan occupancy is not possible in glycomic experiments on limited quantities of cells. It is also possible that changes in *N*-glycosylation are restricted to a subset of glycoproteins and that such subtle differences are lost in the general glycomic profiling. It could also be that the glycosylation in the muscle of the patient is different from that in the cultured cells or the glycosylation might have changed during the use and regeneration of muscle tissue (GFPT1 CMS is later onset than other CMS forms). In addition, the amount of glucose in the medium could influence the glucose flux and subsequently affect the amount of the primary substrate fructose-6-P [9].

The next challenge will be to perform detailed glycoproteomic analysis of specific glycoproteins such as acetylcholine receptor, the muscle-specific kinase (MuSK) and agrin. Indeed similarity between CMS due to *GFPT1* mutations and CMS due to *DPAGT1* mutations would suggest that reduced endplate acetylcholine receptor due to defective *N*-linked glycosylation is a primary disease mechanism in this disorder [3,10,11].

We chose the disease controls, namely CMS caused by mutations in *DOK7*, myopathy caused by mutations in *MTND5*, LGMD2A, and Pompe, as exemplars of muscle diseases which had not previously been found to exhibit defects in *N*-glycosylation. Very recently, however, a study has found that Pompe disease can lead to a Golgi-based glycosylation deficit in human skin fibroblast-derived induced pluripotent stem cells which were differentiated in culture to cardiomyocytes (iPSCCMs) [12]. Using a similar glycomics strategy to ours, Raval *et al.* have shown that there is a reduced diversity of multi-antennary structures and hyposialylation in Pompe iPSCCMs. It should be noted that even their control cells did not show the same high molecular weight multi-antennary *N*-glycans that we observed in this study. It is intriguing that our muscle cells are not showing comparable glycosylation defects. In future work it will be important to investigate whether glycosylation defects in Pompe are cell-specific.

In conclusion we have performed the first detailed *N*-glycan glycomic analysis of myoblasts from healthy control, *GFPT1* patients, a *DOK7* patient, a *MTND5* patient, a LGMD2A patient, and a Pompe disease patient. We also performed detailed structural analysis of *N*-glycans from myotubes derived from a healthy control, a *GFPT1* patient, and a *DOK7* patient. Whilst greatly expanding our knowledge of glycosylations in these cells we did not observe significant changes in disease and control *N*-glycan structures.

## 3. Materials and Methods

### 3.1. Patients

Patients with limb-girdle CMS with tubular aggregates have been reported in two papers [1,2]. Detailed clinical data of the patients was previously published [2]. In brief, the patients presented with fatigable weakness restricted mostly to shoulder and pelvic girdle and minimal or no weakness of facial and ocular muscles. Response to treatment with acetylcholinesterase inhibitors was generally positive. The *DOK7* patient has *DOK7* mutations at c.1124_1127dupTGCC and a deletion of the last 11 bp of exon 4 on cDNA level (second mutation on genomic DNA level not identified), the *MTND5* patient has a *MTND5* mutation at m.13051G>A, the Pompe disease patient has *GAA* mutations at c.-32-13T>G and c.52del (compound heterozygous), the limb girdle muscular dystrophy type 2A patient has CAPN3 mutations at c.1322delG and c.1465C>T (compound heterozygous). Patients and controls were selected at the Newcastle University John Walton Muscular Dystrophy Research Centre, Institute of Genetic Medicine, Newcastle upon Tyne. Their diagnosis was obtained through a combination of clinical and morphological (muscle biopsy) methods and confirmed by genetic testing of the relevant genes. Collection of samples from patients and their use in research have been ethically approved by the NRES Committee North East—Newcastle and North Tyneside 1. All GFPT1 mutations resulted in a reduction of GFPT1 protein amounts in patient muscle. No changes in protein localisation or enzyme activity were observed.

### 3.2. Cell Culture

Primary human skeletal myoblasts of the following patients were analysed: LGM9.3, (GFPT1 mutations p.V199F and c.*22>A), and LGM5.3 and 5.5 (GFPT1 mutations p.M492T and c.*22C>A); patient numbers correspond to numbering in a previous paper [1]. LGM9.3, LGM5.3, and LGM5.5 are GFPT1 patient 1, 2, and 3 respectively. Six control myoblast lines were used, two from healthy individuals and four from diseases unrelated to GFPT1 (mutations in *MTND5*, *DOK7*, *CAPN3*, and *GAA*). Myoblasts from patients and controls were obtained from the MRC Centre for Neuromuscular Diseases Biobank Newcastle, UK, and the Muscle Tissue Culture Collection, Friedrich-Baur-Institute, Munich, Germany. The cells were isolated as previously described [13] and firstly grown in skeletal muscle growth medium (PromoCell, Heidelberg, Germany) supplemented with 5%, 10%, and 15% foetal calf serum. The final concentration of foetal calf serum in the growth medium was 15%. The concentration of glucose in the myoblast growth medium was 1 g/L. Myoblasts were differentiated to myotubes by switching to differentiation medium (DMEM with 2% horse serum) at a cell density of around 80%. The concentration of glucose in the differentiation medium was 4.5 g/L. Cells were grown in differentiation medium for one week.

All studies were carried out with informed consent of the patients or their parents and were approved by institutional ethics review boards.

### 3.3. Processing of Myoblasts and Myotubes to Acquire N- and O-glycans

All myoblast and myotube samples were treated following a standard protocol [14]. Briefly, cells were suspended in lysis buffer (25 mM TRIS, 150 mM NaCl, 5 mM EDTA and 1% CHAPS (v/v), pH 7.4) before homogenisation and sonication were performed. The homogenates were subsequently dialysed against a 50 mM ammonia bicarbonate buffer, pH 7.5, after which the samples were lyophilized. Extracted glycoproteins were reduced and carboxymethylated and digested with trypsin. The digested glycopeptides were purified using a C18 cartridge (Oasis HLB Plus Waters) prior to the release of protein linked *N*-glycans by PNGase F (Roche Applied Science, East Sussex, UK) digest and O-linked glycans by reductive elimination. Released *N*- and *O*-glycans were permethylated and then purified using a Sep-pack C18 cartridge (Waters, Milford, MA, USA) prior to MS analysis. Sialidase cleavage was carried out using sialidase S (Prozyme Glyko, Cambridge, UK) in 50 mM sodium acetate, pH 5.5.

### 3.4. Mass Spectrometric Glycomic Analysis

MS data were obtained via a Voyager MALDI-TOF (Applied Biosystems, Foster City, CA, USA) mass spectrometer. Purified permethylated glycans were dissolved in 10 µL methanol and 1 µL of the sample was mixed with 1 µL of matrix, 20 mg/mL 2,5-dihydroxybenzoic acid (DHB) in in 70% (v/v) aqueous methanol and loaded on to a metal target plate. The instrument was run in the reflectron positive ion mode. The accelerating voltage was 20 kV.

MS/MS data were acquired using a 4800 MALDI-TOF/TOF mass spectrometer (AB SCIEX). In the MS/MS experiment the dissolved sample was dried and then re-dissolved in 10 µL methanol, 1 µL of the sample was mixed with 1 µL of matrix, 10 mg/mL diaminobenzophenone (DABP) in 70% (v/v) aqueous acetonitrile and loaded on to a metal target plate. The instrument was run in the reflectron positive ion mode. The collision energy was set to 1 kV with argon as the collision gas. The 4700 calibration standard (mass standards kit for the 4700 proteomics analyzer, Applied Biosystems) was used as the external calibrant for the MS and MS/MS modes.

### 3.5. Analyses of MALDI Data

The MS and MS/MS data were processed employing Data Explorer Software from Applied Biosystems. The processed spectra were annotated using a glycobioinformatics tool, GlycoWorkBench [15]. Based on known biosynthetic pathways and susceptibility to PNGase F digestion, all *N*-glycans are presumed to have a Manα1–6(Manα1–3)Manβ1–4GlcNAcβ1–4GlcNAc core structure [16,17]. The symbolic nomenclature used in the spectra annotation is the same as the one used by the Consortium for Functional Glycomics (CFG) (http://www.functionalglycomics.org/) and the Essentials for Glycobiology on-line textbook (http://www.ncbi.nlm.nih.gov/books/NBK1931/figure/ch1.f5/?report=objectonl). Reproducibility and comparability were assessed by ANOVA (see Figure S5).

## 4. Conclusions

The glycomic analysis of myoblasts reveals that the patterns of branching and/or antennae extensions are very similar amongst the myoblasts from *GFPT1* patients, healthy controls, and the other muscle disease patients studied.

In addition, analysis of myotubes reveals that there is no significant difference in the MALDI profiles from *GFPT1* patients, healthy controls, and the other muscle disease patients studied.

## Supplementary Files

Supplementary File 1

## References

[1] Senderek J., Muller J.S., Dusl M., Strom T.M., Guergueltcheva V., Diepolder I., Laval S.H., Maxwell S., Cossins J., Krause S. (2011). Hexosamine biosynthetic pathway mutations cause neuromuscular transmission defect. Am. J. Hum. Genet..

[2] Guergueltcheva V., Muller J.S., Dusl M., Senderek J., Oldfors A., Lindbergh C., Maxwell S., Colomer J., Mallebrera C.J., Nascimento A. (2011). Congenital myasthenic syndrome with tubular aggregates caused by GFPT1 mutations. J. Neurol..

[3] Belaya K., Finlayson S., Slater C.R., Cossins J., Liu W.W., Maxwell S., McGowan S.J., Maslau S., Twigg S.R., Walls T.J. (2012). Mutations in DPAGT1 cause a limb-girdle congenital myasthenic syndrome with tubular aggregates. Am. J. Hum. Genet..

[4] Cossins J., Belaya K., Hicks D., Salih M.A., Finlayson S., Carboni N., Liu W.W., Maxwell S., Zoltowska K., Farsani G.T. (2013). Congenital myasthenic syndromes due to mutations in ALG2 and ALG14. Brain.

[5] Monk C.R., Sutton-Smith M., Dell A., Garden O.A. (2006). Preparation of CD25(+) and CD25(−) CD4(+) T cells for glycomic analysis—A cautionary tale of serum glycoprotein sequestration. Glycobiology.

[6] Dell A., Morris H.R. (2001). Glycoprotein structure determination mass spectrometry. Science.

[7] Antonopoulos A., Demotte N., Stroobant V., Haslam S.M., van der Bruggen P., Dell A. (2012). Loss of effector function of human cytolytic T lymphocytes is accompanied by major alterations in *N*- and *O*-glycosylation. J. Biol. Chem..

[8] Sassi A., Lazaroski S., Wu G., Haslam S.M., Fliegauf M., Mellouli F., Patiroglu T., Unal E., Ozdemir M.A., Jouhadi Z. (2014). Hypomorphic homozygous mutations in phosphoglucomutase 3 (PGM3) impair immunity and increase serum IGE levels. J. Allergy Clin. Immunol..

[9] Schleicher E.D., Weigert C. (2000). Role of the hexosamine biosynthetic pathway in diabetic nephropathy. Kidney Int. Suppl..

[10] Zoltowska K., Webster R., Finlayson S., Maxwell S., Cossins J., Muller J., Lochmuller H., Beeson D. (2013). Mutations in GFPT1 that underlie limb-girdle congenital myasthenic syndrome result in reduced cell-surface expression of muscle AChR. Hum. Mol. Genet..

[11] Freeze H.H., Eklund E.A., Ng B.G., Patterson M.C. (2015). Neurological aspects of human glycosylation disorders. Annu. Rev. Neurosci..

[12] Raval K.K., Tao R., White B.E., de Lange W.J., Koonce C.H., Yu J., Kishnani P.S., Thomson J.A., Mosher D.F., Ralphe J.C. (2015). Pompe disease results in a GOLGI-based glycosylation deficit in human induced pluripotent stem cell-derived cardiomyocytes. J. Biol. Chem..

[13] Lochmuller H., Johns T., Shoubridge E.A. (1999). Expression of the E6 and E7 genes of human papillomavirus (HPV16) extends the life span of human myoblasts. Exp. Cell Res..

[14] Jang-Lee J., North S.J., Sutton-Smith M., Goldberg D., Panico M., Morris H., Haslam S., Dell A. (2006). Glycomic profiling of cells and tissues by mass spectrometry: Fingerprinting and sequencing methodologies. Methods Enzymol..

[15] Ceroni A., Maass K., Geyer H., Geyer R., Dell A., Haslam S.M. (2008). Glycoworkbench: A tool for the computer-assisted annotation of mass spectra of glycans. J. Proteome Res..

[16] Schachter H. (1991). The “yellow brick road” to branched complex *N*-glycans. Glycobiology.

[17] North S.J., Jang-Lee J., Harrison R., Canis K., Ismail M.N., Trollope A., Antonopoulos A., Pang P.C., Grassi P., Al-Chalabi S. (2010). Mass spectrometric analysis of mutant mice. Methods Enzymol..

