# Supplementary material for: Global N-linked Glycosylation is Not Significantly Impaired in Myoblasts in Congenital Myasthenic Syndromes Caused by Defective Glutamine-Fructose-6-Phosphate Transaminase 1 (GFPT1)
Source: Biomolecules. 2015 Oct 16;5(4):2758–81. doi: 10.3390/biom5042758 (PMC4693256; doi:10.3390/biom5042758)
Supplement: Supplementary File 1 [file biomolecules-05-02758-s001.pdf]

Supplementary Materials

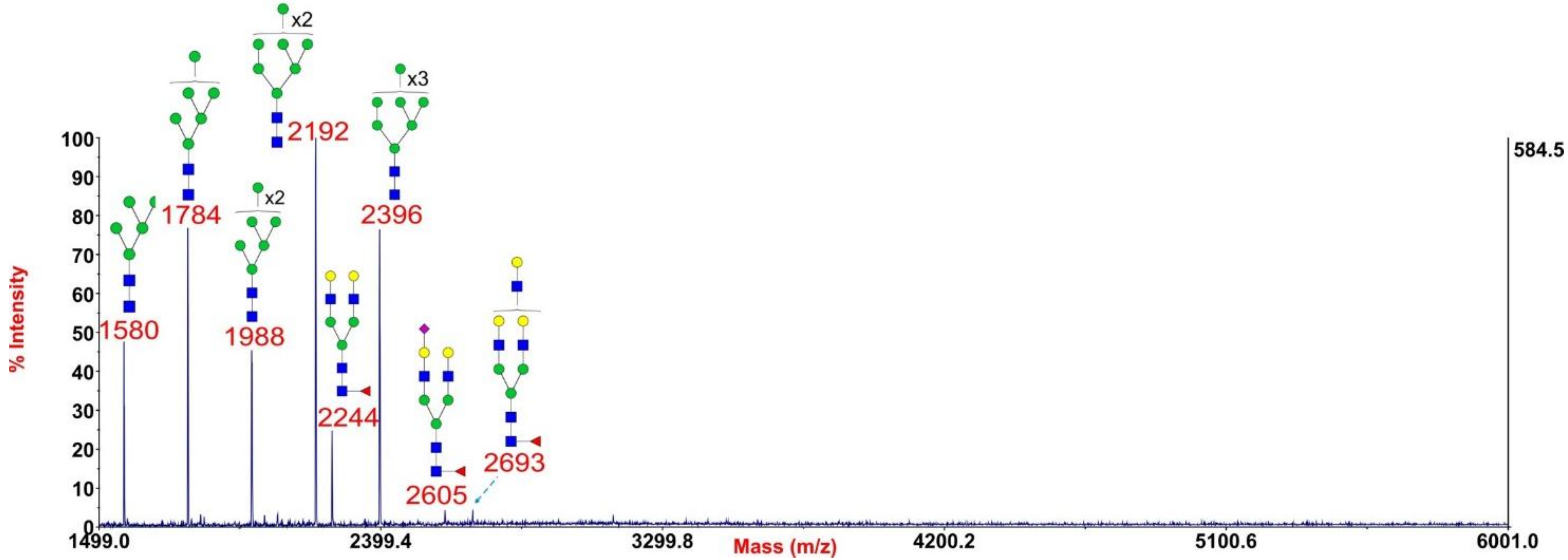

(**A**)

Figure S1. *Cont*.

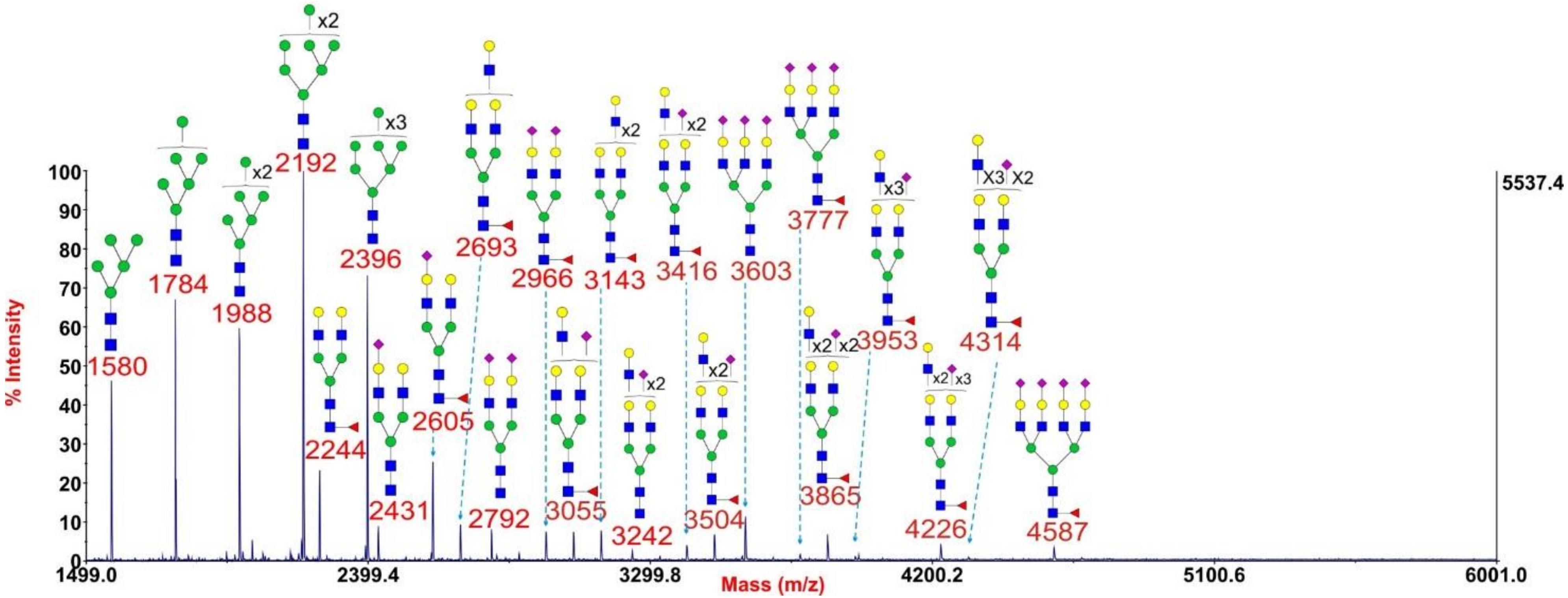

(B)

Figure S1. *Cont.*

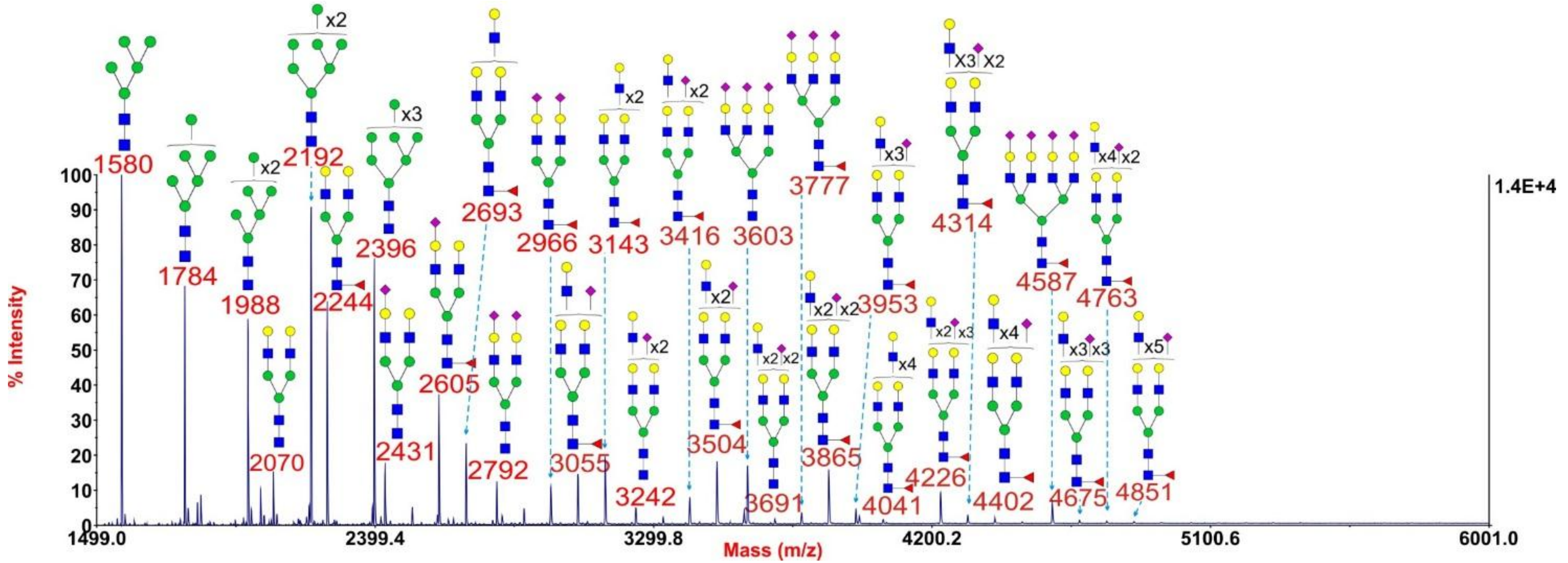

(**C**)

Figure S1. Annotated MALDI-TOF MS spectra of permethylated N-glycans from myoblasts of the CMS patient caused by mutations in *DOK7*, the myoblasts were cultured in the medium containing 5% (**A**); 10% (**B**) and 15% (**C**) FCS Profiles were obtained from the 50% acetonitrile fraction from a C18 Sep-Pak column. All ions are $[M + Na]^+$. The number indicated in the spectra is the mass to charge ratio (m/z) of the corresponding ion. Since the ion is monocharged, the value of m/z is equal to the molecular weight value of the glycan. Annotations are based on the molecular weight, *N*-glycan biosynthetic pathway and MS/MS data. Glycans at m/z 2966, 3777 and 4587 are clearly annotated, this is due to the fact that their structures are unequivocal because each antenna is capped with a sialic acid and thus they are homogeneous bi-, tri- and tetraantennary glycans. However, the glycan structure is not always as unequivocal as the glycan at m/z 2966 as biosynthetically non-fully sialylated glycan molecular ion species could be made up of mixtures of structural isoforms. Therefore, for those heterogeneous multiantennary structures with extended LacNAc repeats, the annotations are simplified throughout by using biantennary structures with the extensions and NeuAcs listed outside a bracket. ■ GlcNAc, ● Man, ● Gal, ▲ Fuc, ◆ NeuAc.

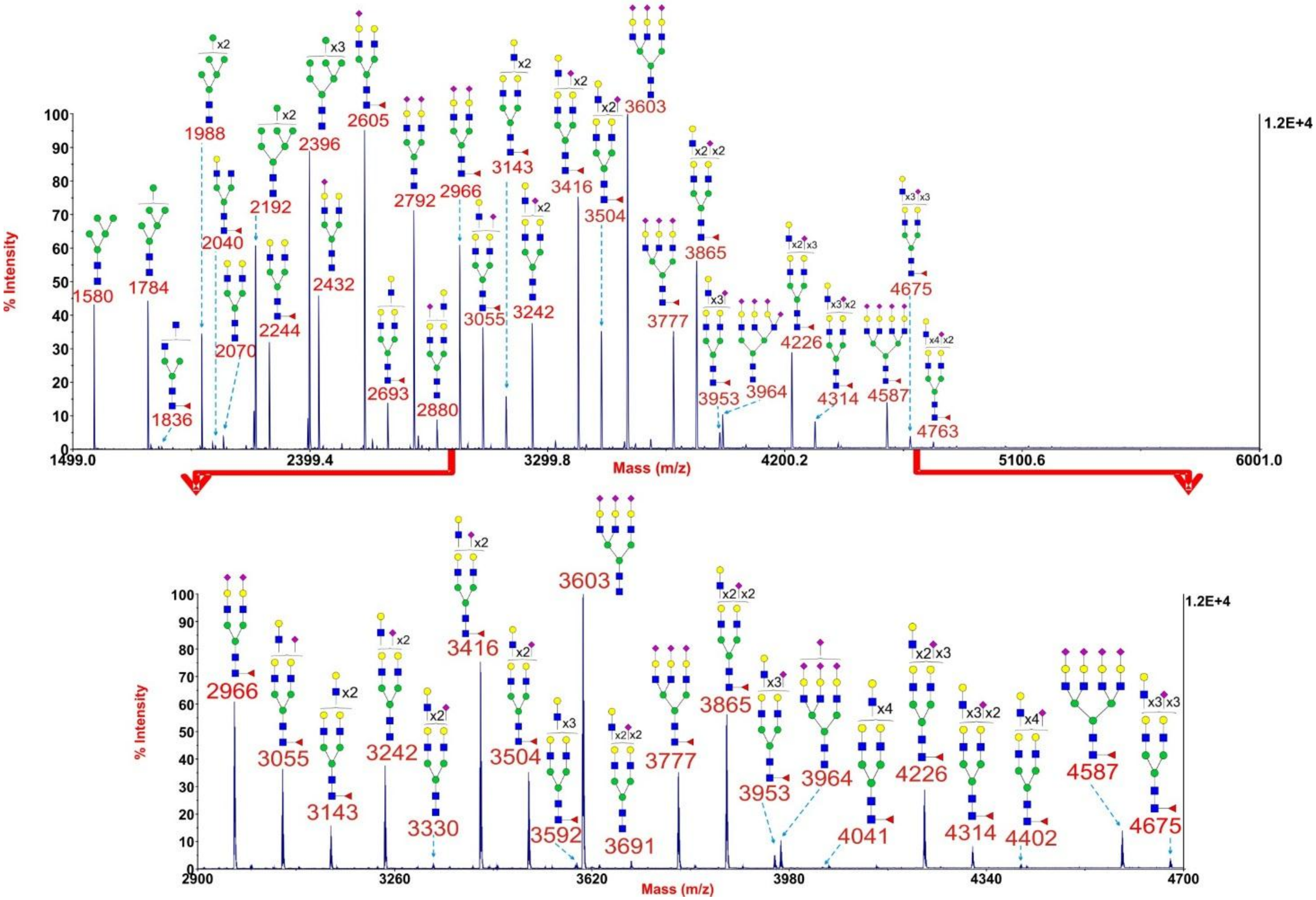

(**A**)

Figure S2. *Cont.*

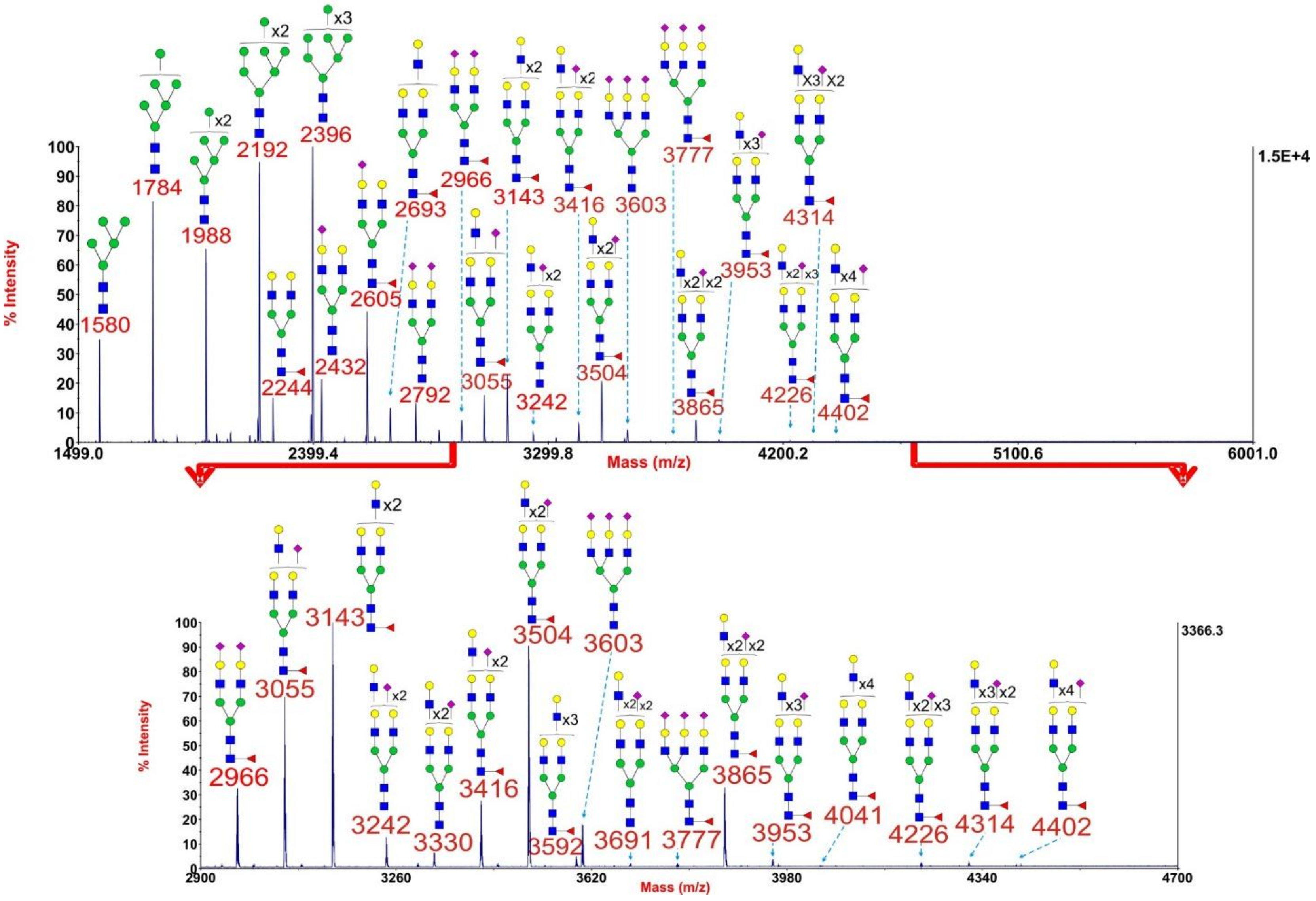

(**B**)

Figure S2. *Cont.*

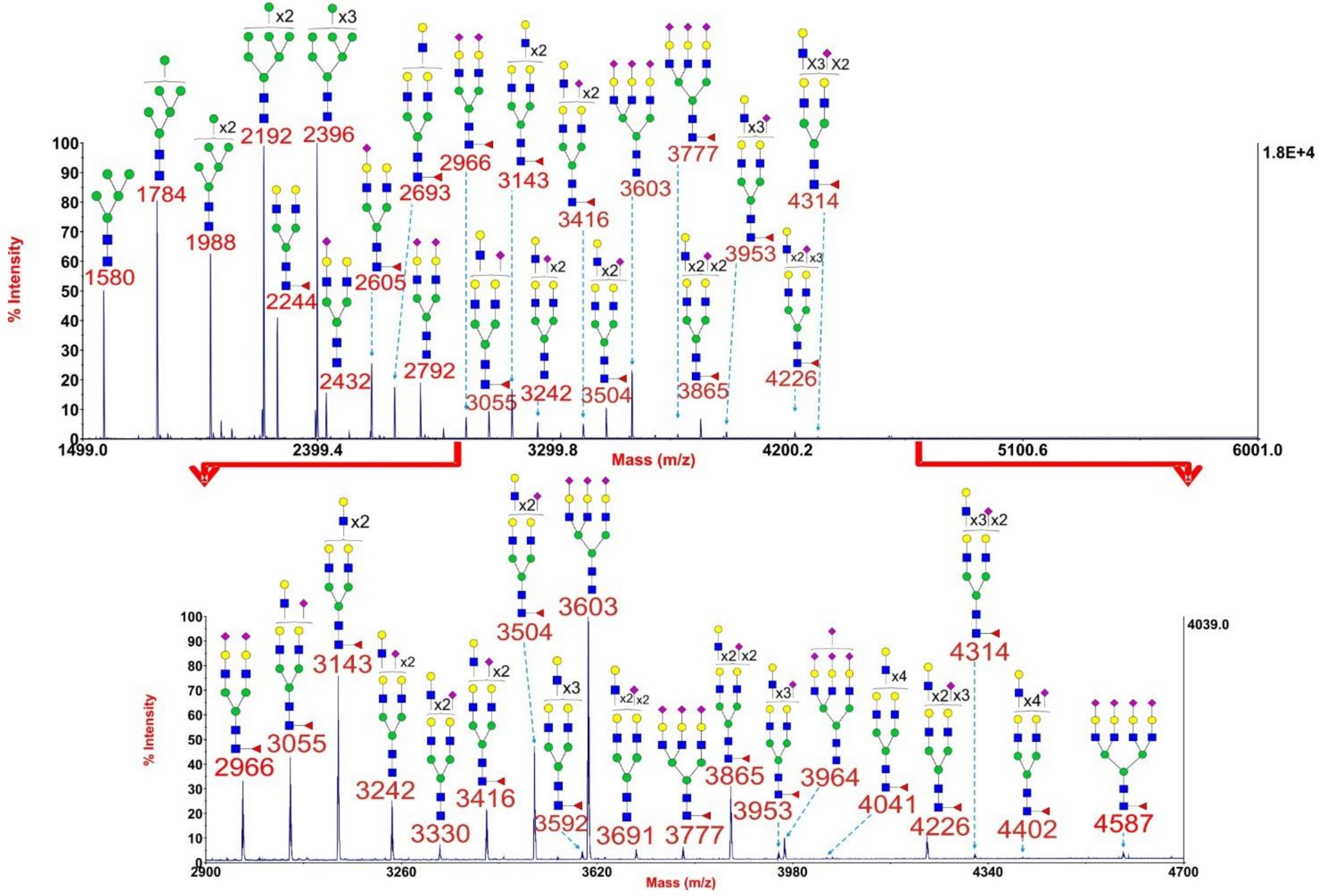

(**C**)

Figure S2. *Cont.*

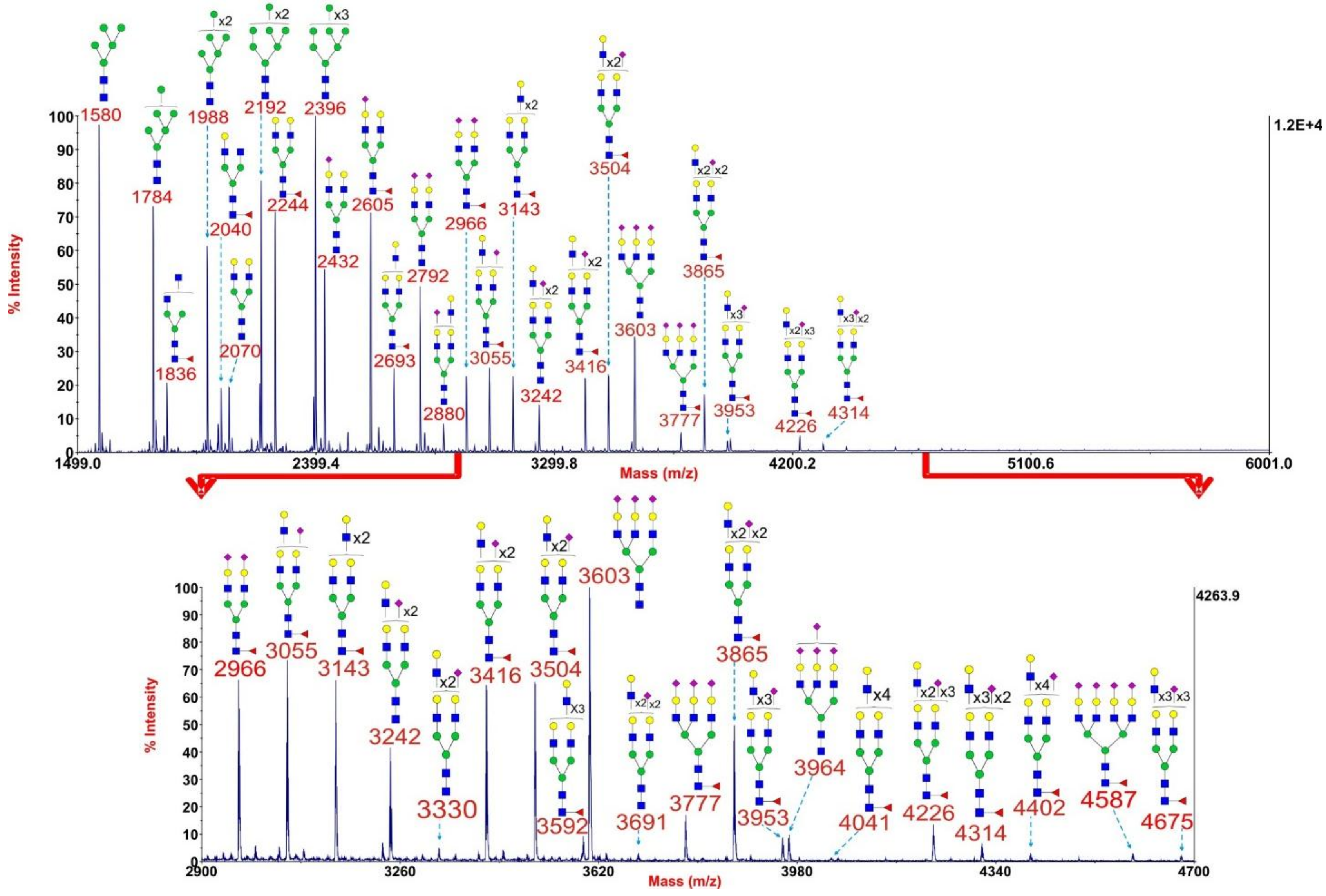

(**D**)

Figure S2. *Cont.*

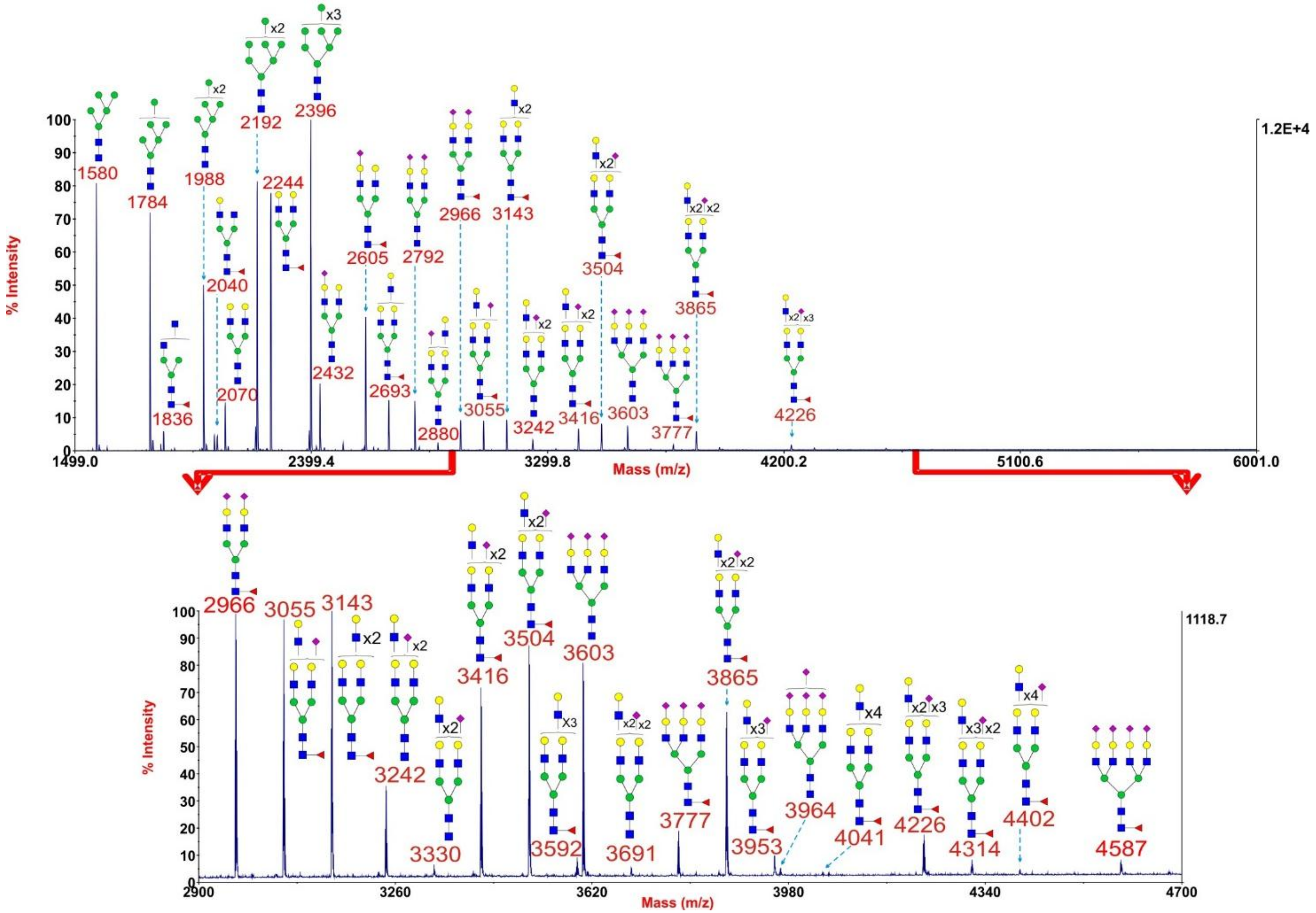

(**E**)

Figure S2. Annotated MALDI-TOF MS spectra of permethylated *N*-glycans from myoblasts of healthy control 2 (**A**); *GFPT1* patient 2 (**B**); the *MTND5* patient (**C**); limb girdle muscular dystrophy type 2A (LGMD2A) patient (**D**) and Pompe disease patient (**E**) In each of A, B, C, D and E, the top panel shows the full spectrum of glycans and the bottom panel amplifies the mass range where the majority of tri- and tetra-antennary

glycans are found, the starting point and ending point of which have been indicated by red arrows. Profiles were obtained from the 50% acetonitrile fraction from a C18 Sep-Pak column. All ions are $[M + Na]^+$. The number indicated in the spectra is the mass to charge ratio (m/z) of the corresponding ion. Since the ion is monocharged, the value of m/z is equal to the molecular weight value of the glycan. Annotations are based on the molecular weight, *N*-glycan biosynthetic pathway and MS/MS data. Glycans at m/z 2966, 3777 and 4587 are clearly annotated, this is due to the fact that their structures are unequivocal because each antenna is capped with a sialic acid and thus they are homogeneous bi-, tri- and tetraantennary glycans. However, the glycan structure is not always as unequivocal as the glycan at m/z 2966 as biosynthetically non-fully sialylated glycan molecular ion species could be made up of mixtures of structural isoforms. Therefore, for those heterogeneous multiantennary structures with extended LacNAc repeats, the annotations are simplified throughout by using biantennary structures with the extensions and NeuAcs listed outside a bracket. ■ GlcNAc, ● Man, ○ Gal, ▲ Fuc, ◆ NeuAc.

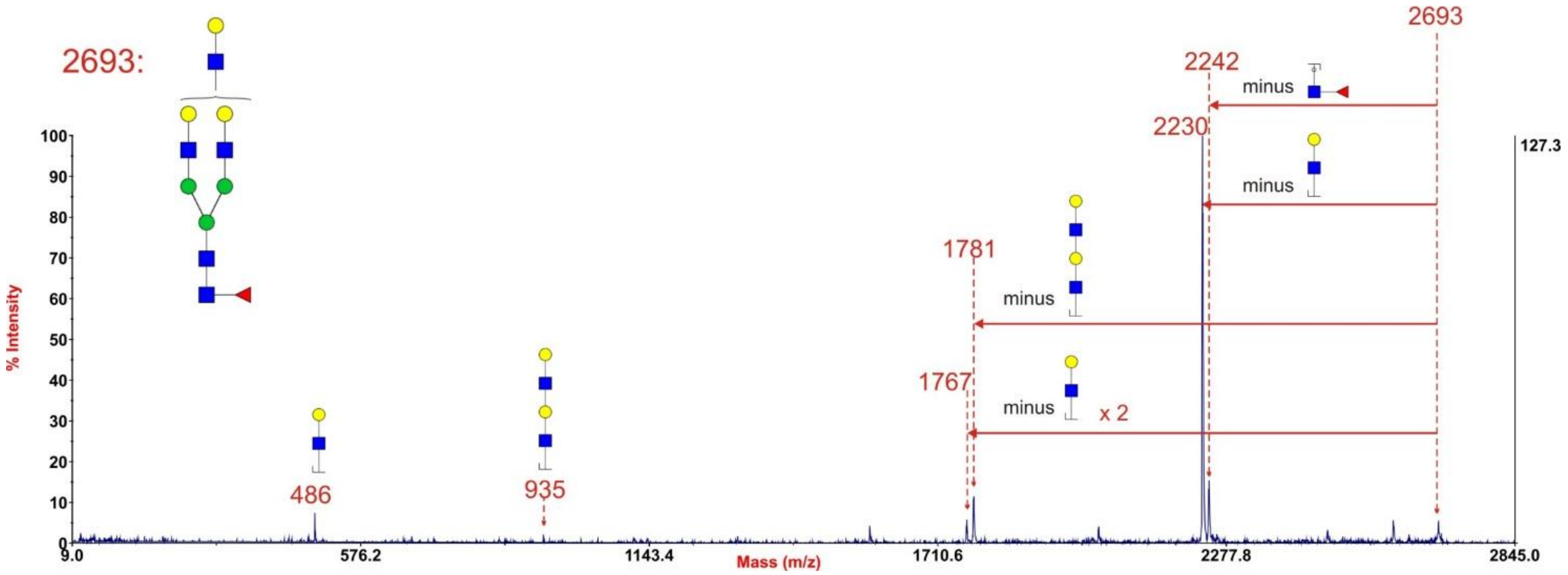

(A)

Figure S3. *Cont.*

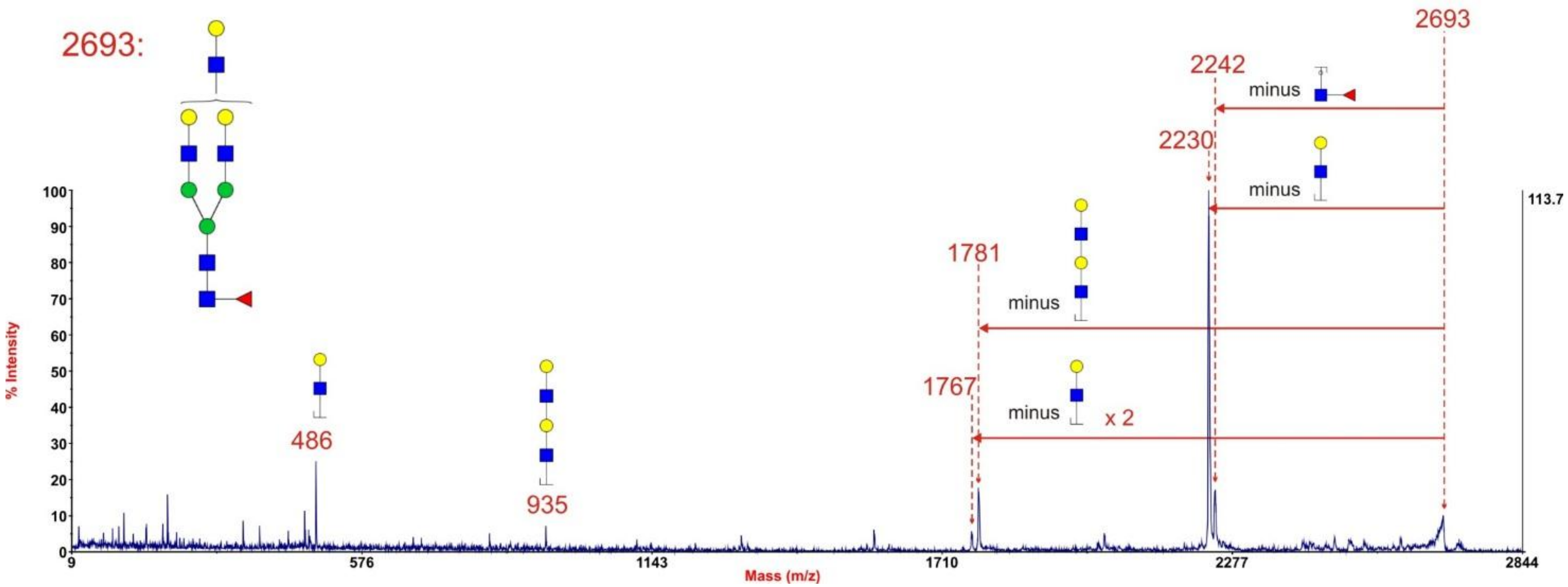

(**B**)

Figure S3. *Cont.*

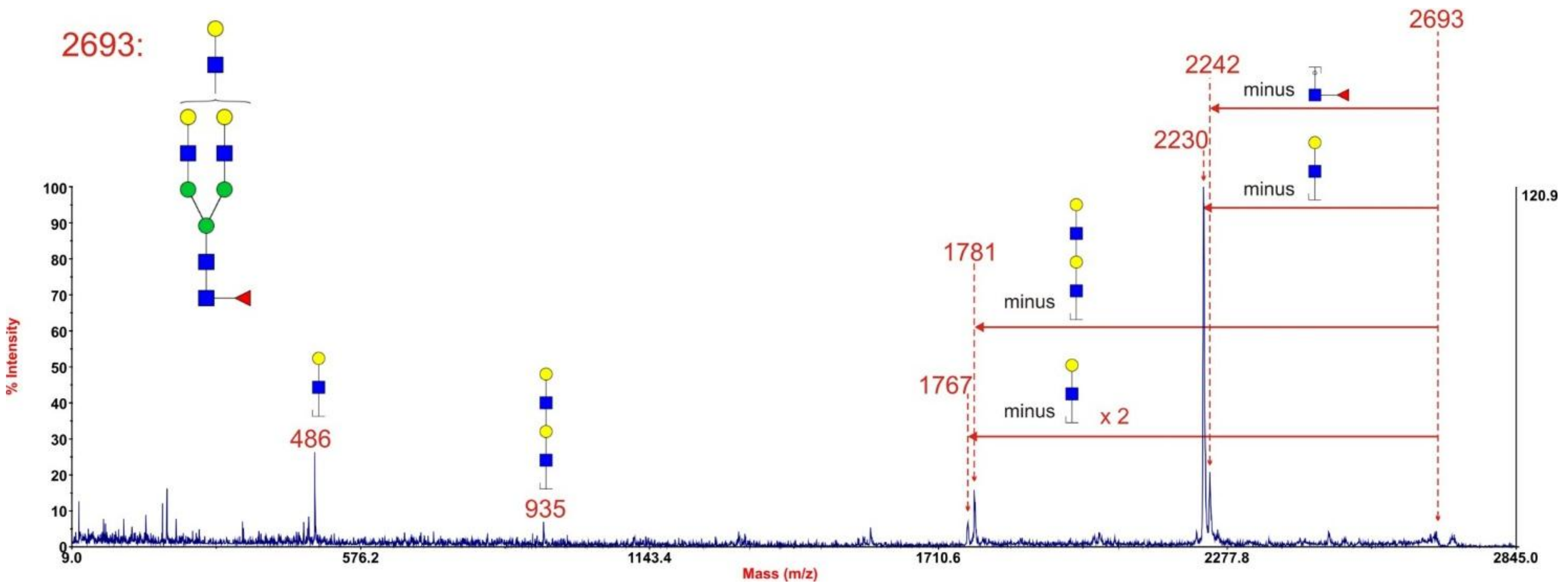

(**C**)

Figure S3. MALDI-TOF/TOF MS/MS spectra of the permethylated *N*-glycan at m/z 2693 $[M + Na]^+$ in myoblasts from healthy control 1 (**A**); *GFPT1* patient 1 (**B**) and the *DOK7* patient (**C**) This glycan is a non-sialylated glycan with three lacNAc units (m/z 2693) in the MALDI data (see Figure 1). Two antennae arrangements are consistent with this composition: triantennary and/or biantennary with one LacNAc extension. The MS/MS spectra are dominated by the fragment ion arising from loss of a single terminal LacNAc, this fragment ion can be derived from both the bi-and tri-antennary options. Nevertheless there are several fragment ions that are diagnostic for the extended biantennary structure. These are observed at m/z 935 and 1781. Importantly their abundances relative to the major fragment ions are similar in the three samples. We estimate from abundance comparisons that m/z 2693 is comprised of about 91% tri-antennary and 9% extended bi-antennary structures in healthy control 1, approximately 86% tri-antennary and 14% extended bi-antennary structures in GFPT1 patient 1, and roughly 88% tri-antennary and 12% extended bi-antennary structures in the DOK7 patient. Assignments of the fragment ions are indicated on the cartoons and on the spectra the horizontal red arrows show antennae losses whilst antennae-derived fragment ions are annotated with their sequences. ■ GlcNAc, ● Man, ● Gal, ▲ Fuc, ◆ NeuAc.

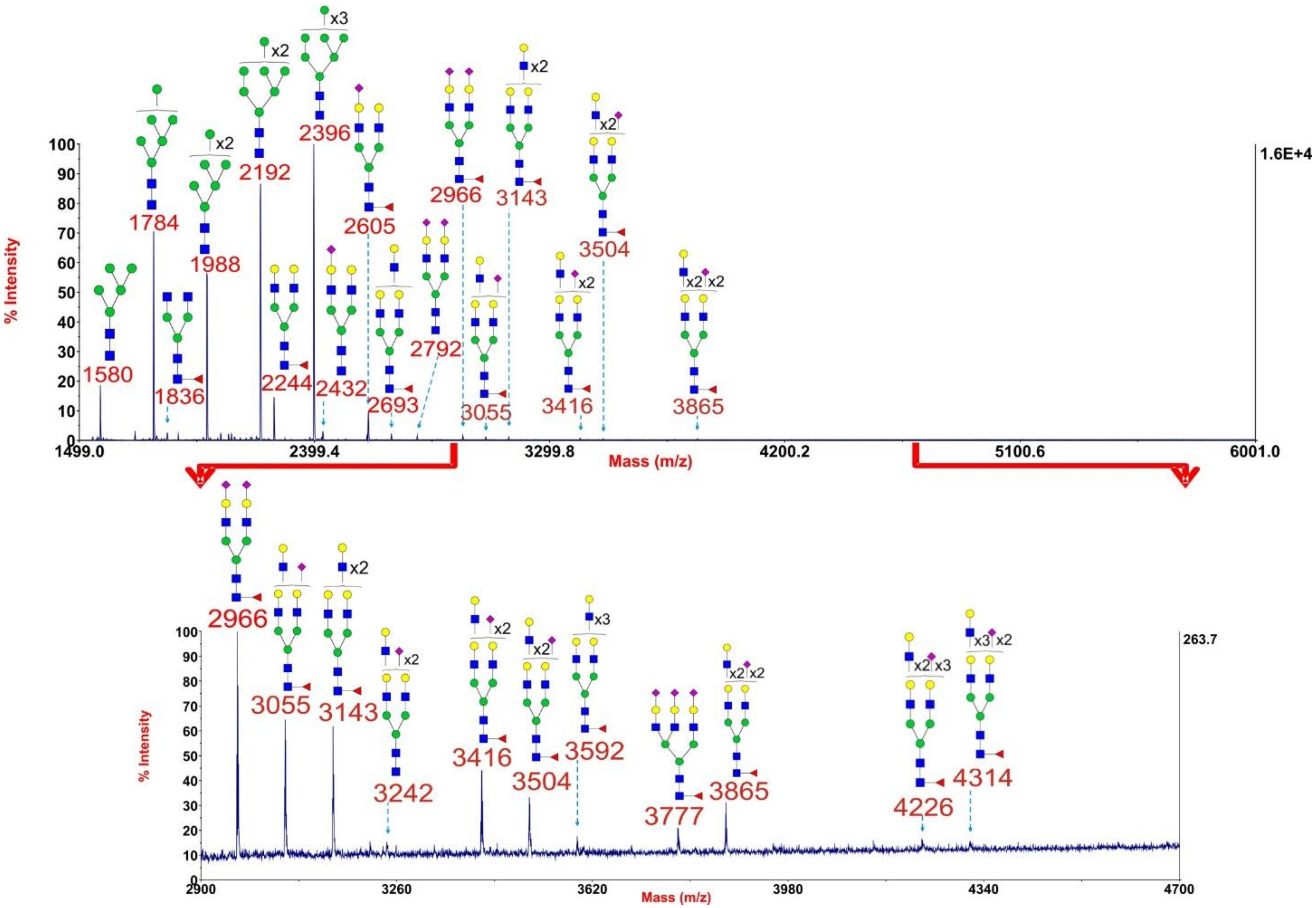

(A)

Figure S4. *Cont.*

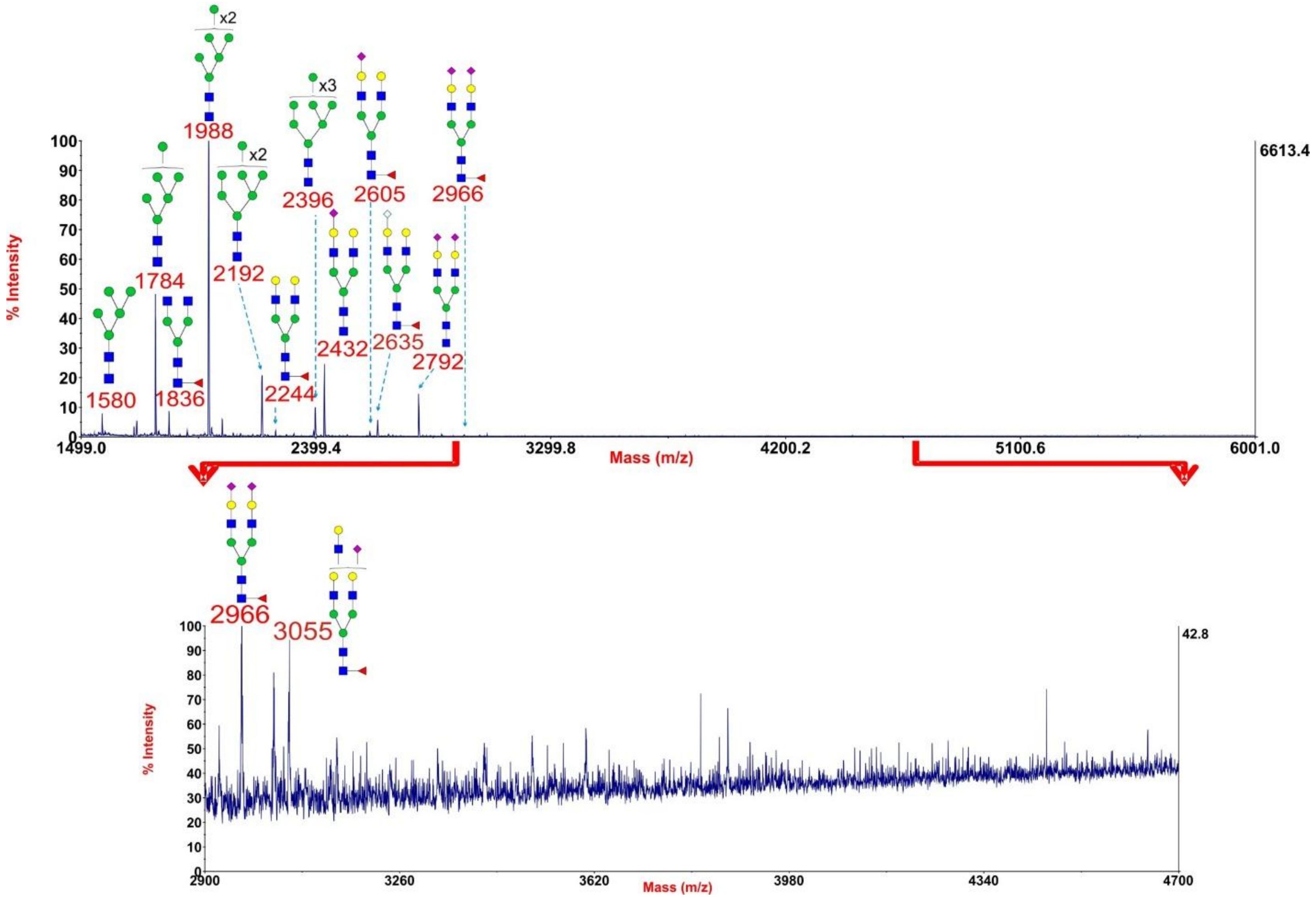

(**B**)

Figure S4. *Cont.*

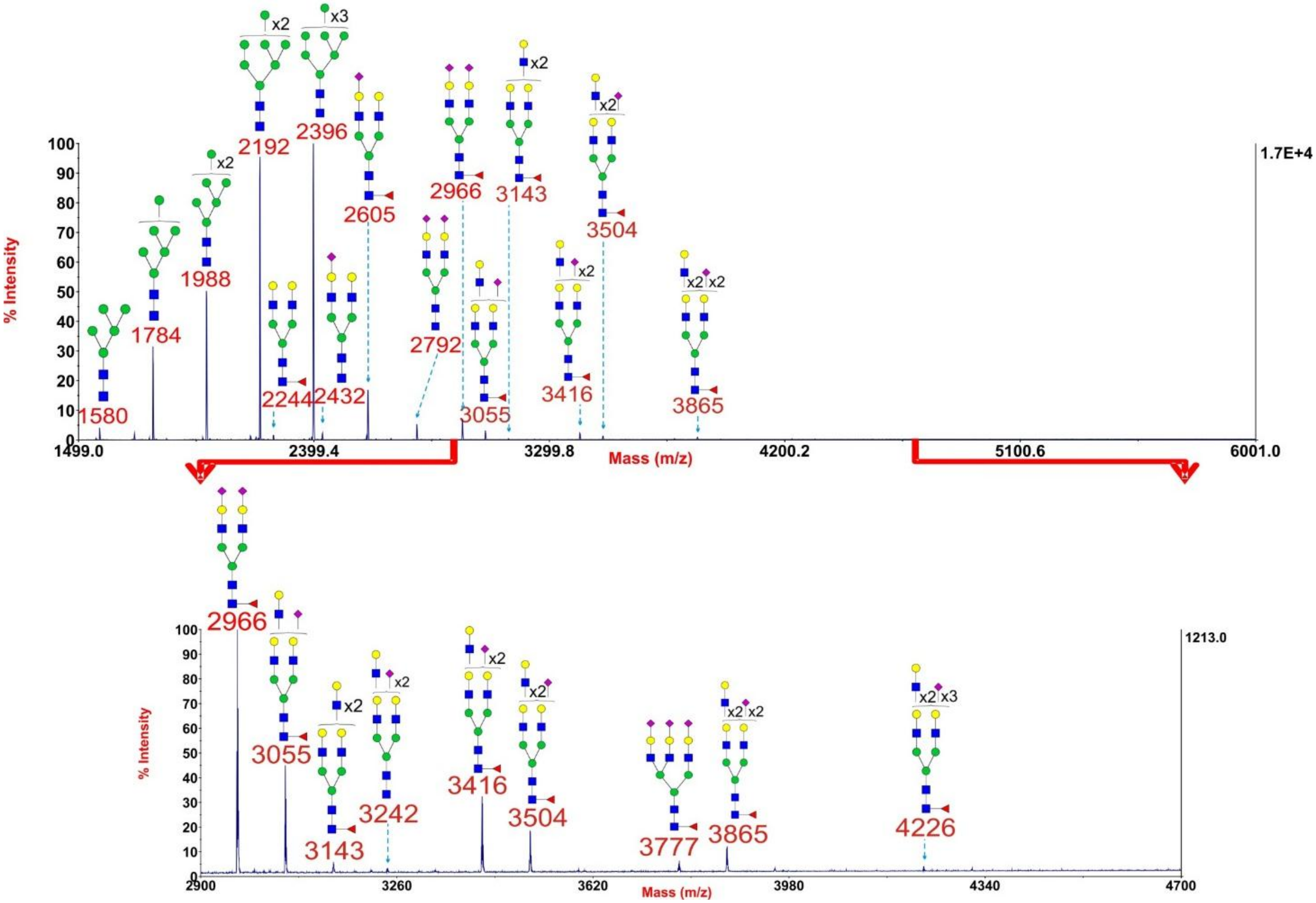

(**C**)

Figure S4. *Cont.*

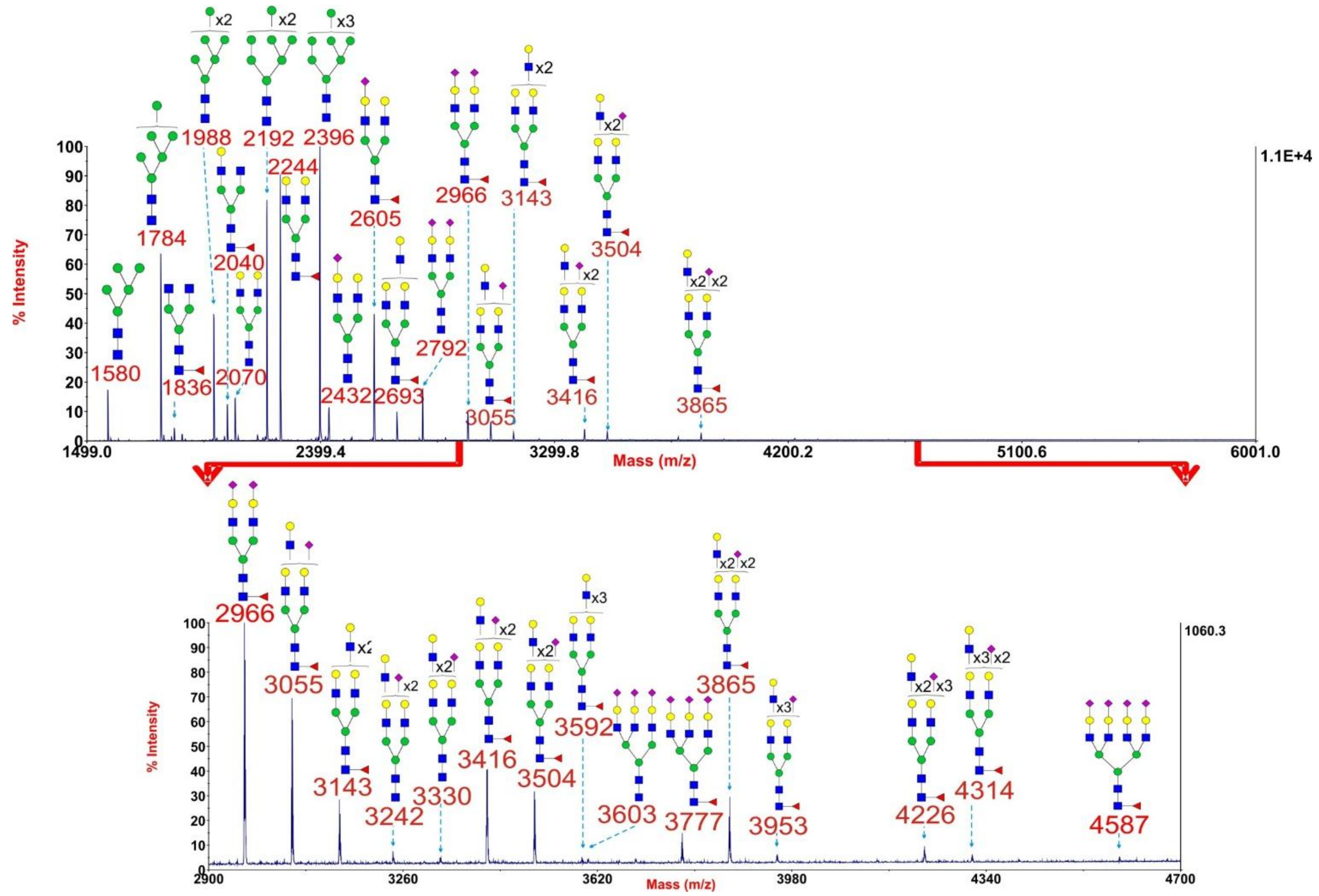

(**E**)

Figure S4. Annotated MALDI-TOF MS spectra of permethylated *N*-glycans from myotubes of healthy control 2 (**A**); *GFPT1* patient 2 (**B**); the *MTND5* patient (**C**); limb girdle muscular dystrophy type 2A (LGMD2A) patient (**D**) and Pompe disease patient (**E**) In each of A, B, C, D and E, the top panel shows the full spectrum of glycans and the bottom panel amplifies the mass range where the majority of tri- and tetra-antennary glycans are found, the starting point and ending point of which have been indicated by red arrows. Profiles were obtained from the 50% acetonitrile fraction from a C18 Sep-Pak column. All ions are $[M + Na]^+$. The number indicated in the spectra is the mass to charge ratio (m/z)

of the corresponding ion. Since the ion is monocharged, the value of m/z is equal to the molecular weight value of the glycan. Annotations are based on the molecular weight, *N*-glycan biosynthetic pathway and MS/MS data. Glycans at m/z 2966, 3777 and 4587 are clearly annotated, this is due to the fact that their structures are unequivocal because each antenna is capped with a sialic acid and thus they are homogeneous bi-, tri- and tetraantennary glycans. However, the glycan structure is not always as unequivocal as the glycan at m/z 2966 as biosynthetically non-fully sialylated glycan molecular ion species could be made up of mixtures of structural isoforms. Therefore, for those heterogeneous multiantennary structures with extended LacNAc repeats, the annotations are simplified throughout by using biantennary structures with the extensions and NeuAcs listed outside a bracket. ■ GlcNAc, ● Man, ○ Gal, ▲ Fuc, ◆ NeuAc.

Healthy control 1

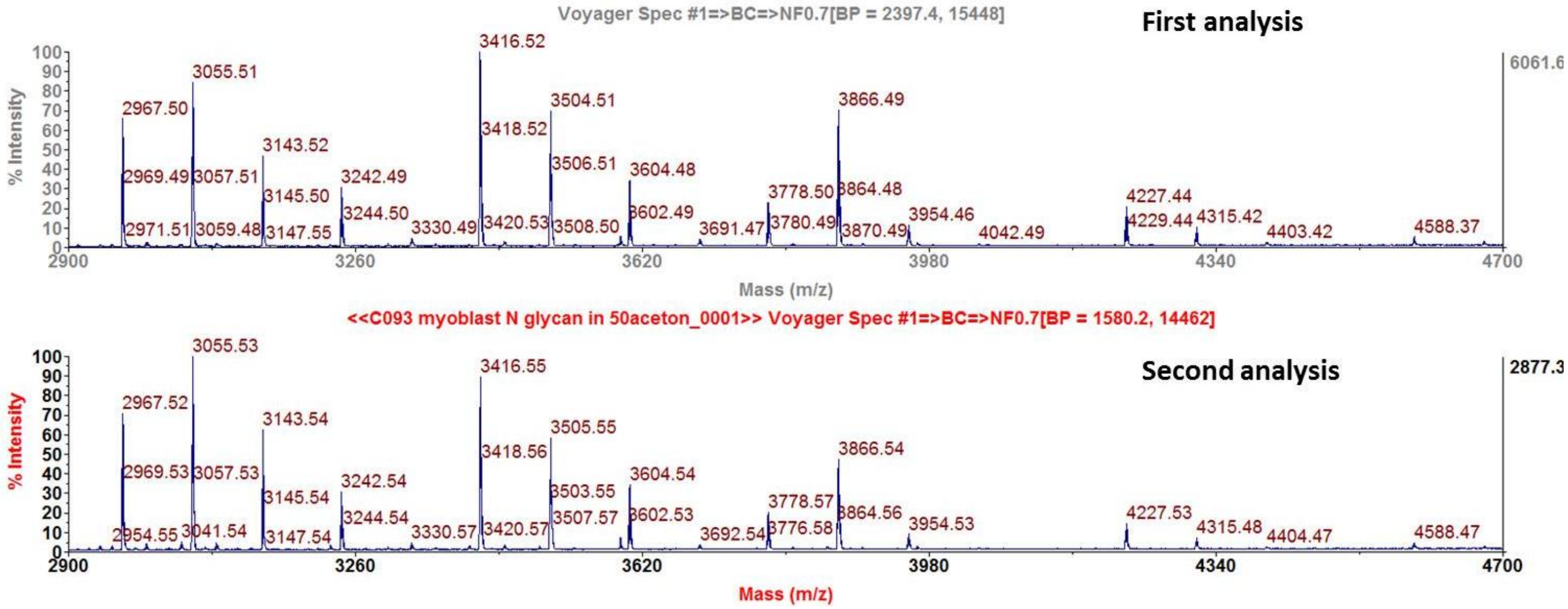

(A)

Figure S5. *Cont.*

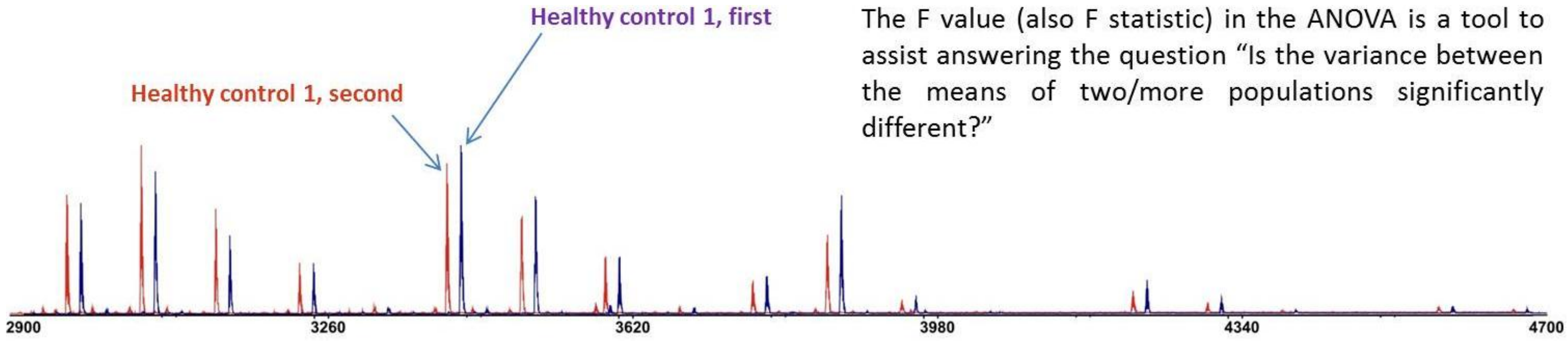

The F value (also F statistic) in the ANOVA is a tool to assist answering the question "Is the variance between the means of two/more populations significantly different?"

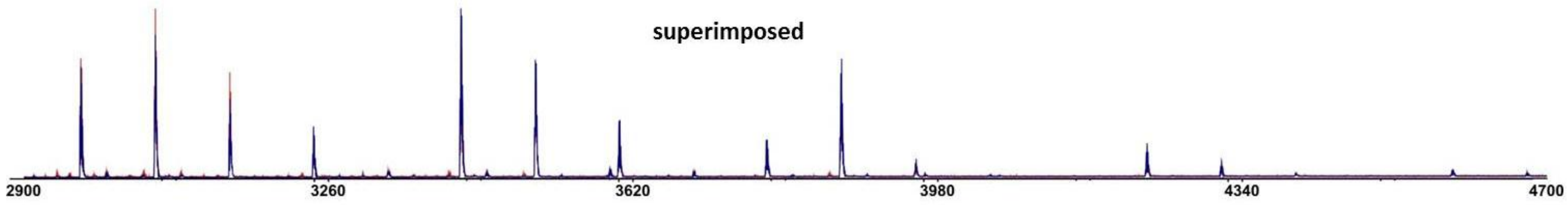

SUMMARY							
Groups	Count	Sum	Average	Variance			
myoblast healthy control 1, first analysis	13	5.1361	0.3951	0.1203			
myoblast healthy control 1, second analysis	13	4.9222	0.3786	0.1228			
ANOVA							
Source of Variation	SS	df	MS	F	P-value	F critical	
Between Groups	0.00176	1	0.0018	0.0145	0.9052	4.2597	
Within Groups	2.91668	24	0.1215				
Total	2.91844	25					

The alpha factor chosen was is 0.05.

As shown in the table the F value is much smaller than the F critical value, indicating that there is no significant difference between the two groups; thus the data are reproducible.

It should be noted that non-core-fucosylated N-glycans, such as m/z 3603 and 3242 were not involved in this comparison as they were derived from contaminating foetal calf serum (see Results).

(**B**)

Figure S5. *Cont.*

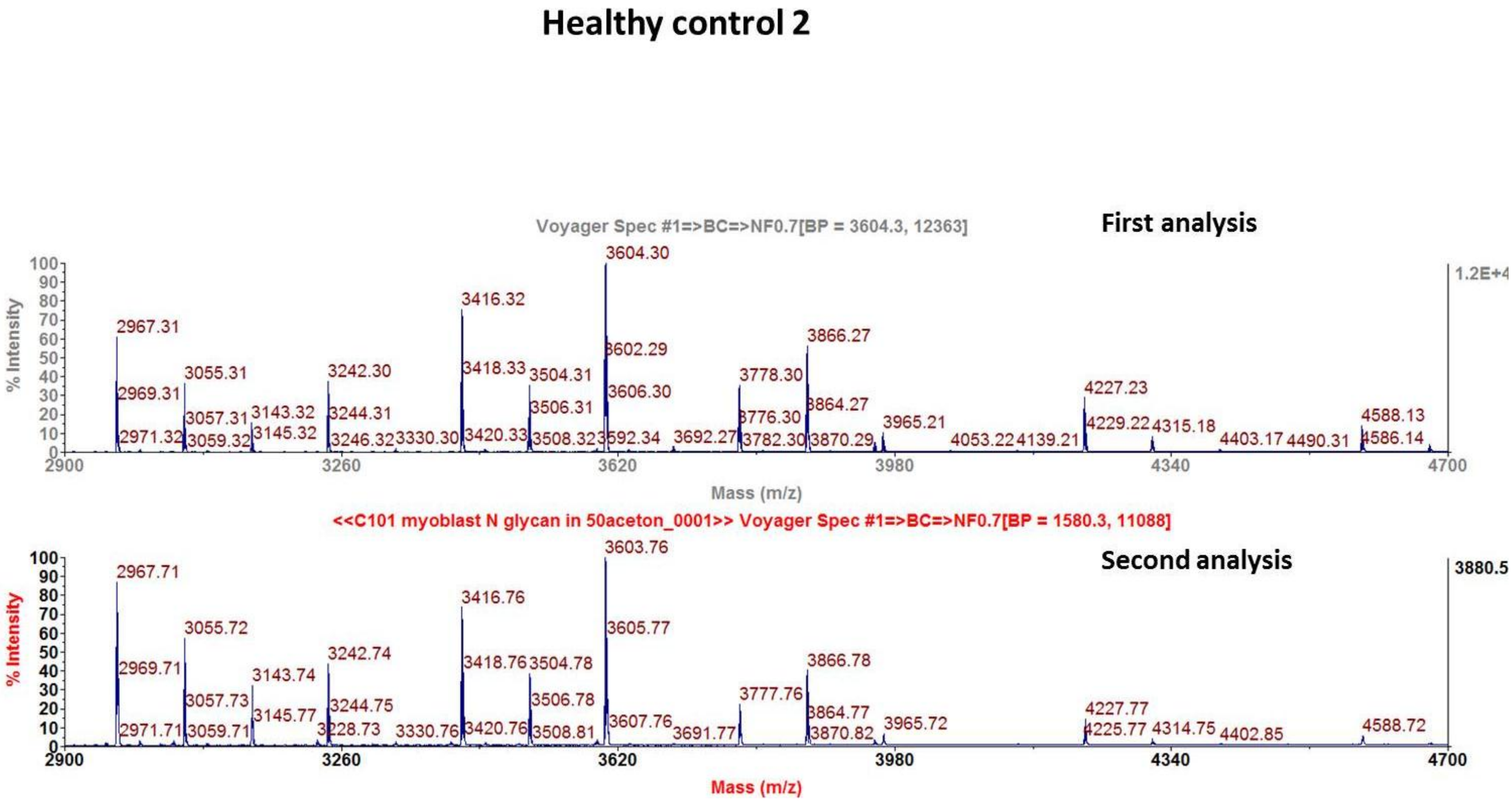

(**C**)

Figure S5. *Cont.*

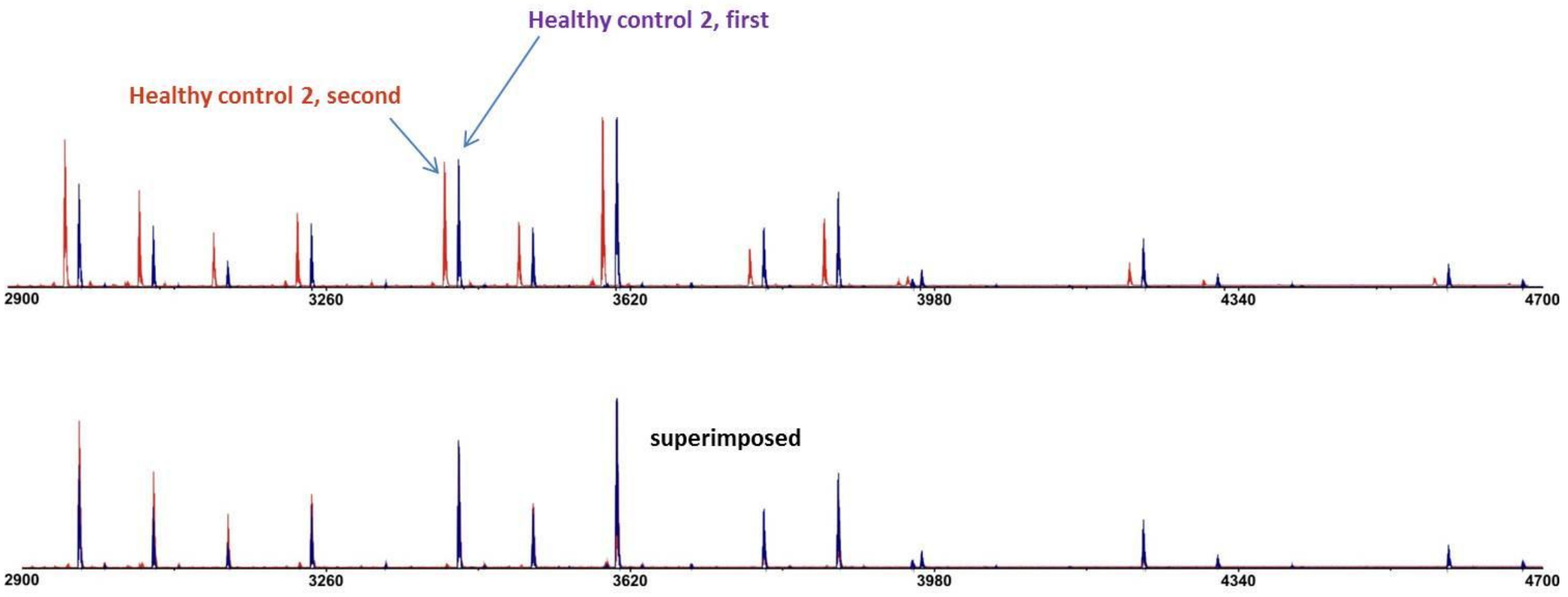

SUMMARY							
Groups	Count	Sum	Average	Variance			
myoblast healthy control 2, first analysis	14	3.758	0.268	0.0586			
myoblast healthy control 2, first analysis	14	3.804	0.272	0.0828			
ANOVA							
Source of Variation	SS	df	MS	F	P-value	F critical	
Between Groups	8E-05	1	8E-05	0.0011	0.974	4.225	
Within Groups	1.838	26	0.071				
Total	1.838	27					

(**D**)

Figure S5. *Cont.*

***GFPT1* patient 1**

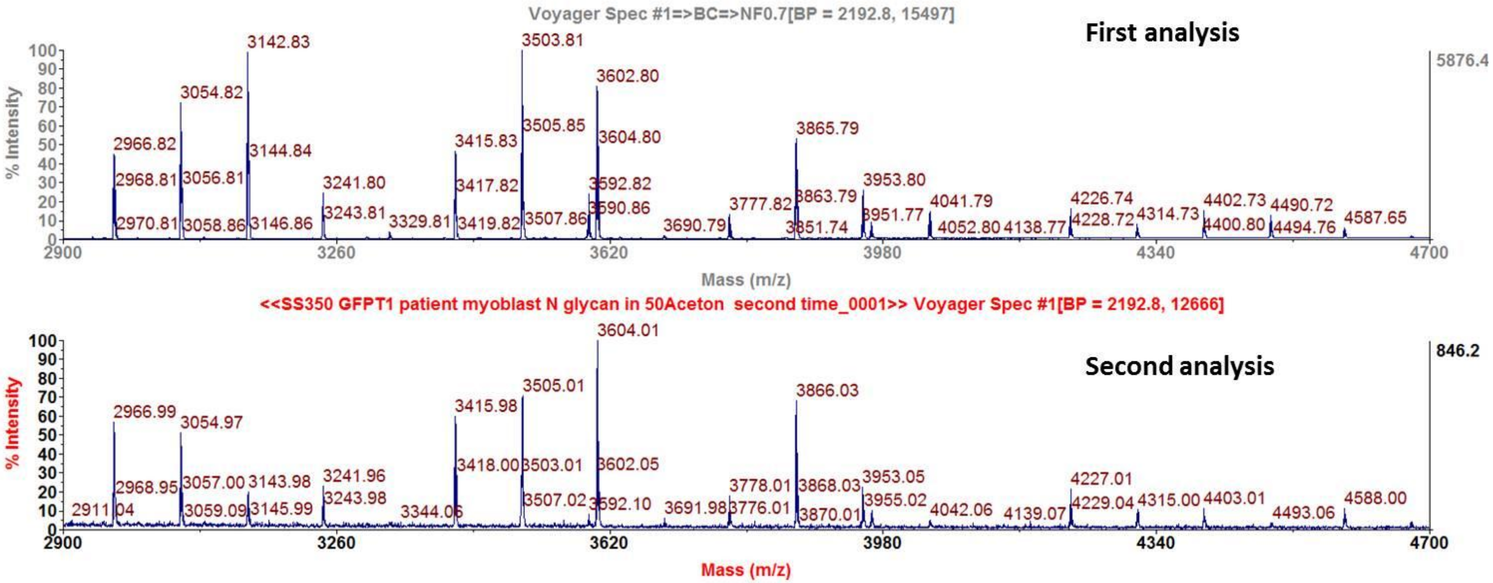

Anova: Single Factor							
SUMMARY							
Groups	Count	Sum	Average	Variance			
GFPT1 patient 1, first analysis	14	5.4019	0.3858	0.1034			
GFPT1 patient 1, second analysis	14	4.3793	0.3128	0.0571			
ANOVA							
Source of Variation	SS	df	MS	F	P-value	F critical	
Between Groups	0.03735	1	0.0373	0.4656	0.5011	4.2252	
Within Groups	2.08577	26	0.0802				
Total	2.12312	27					

(E)

Figure S5. *Cont.*

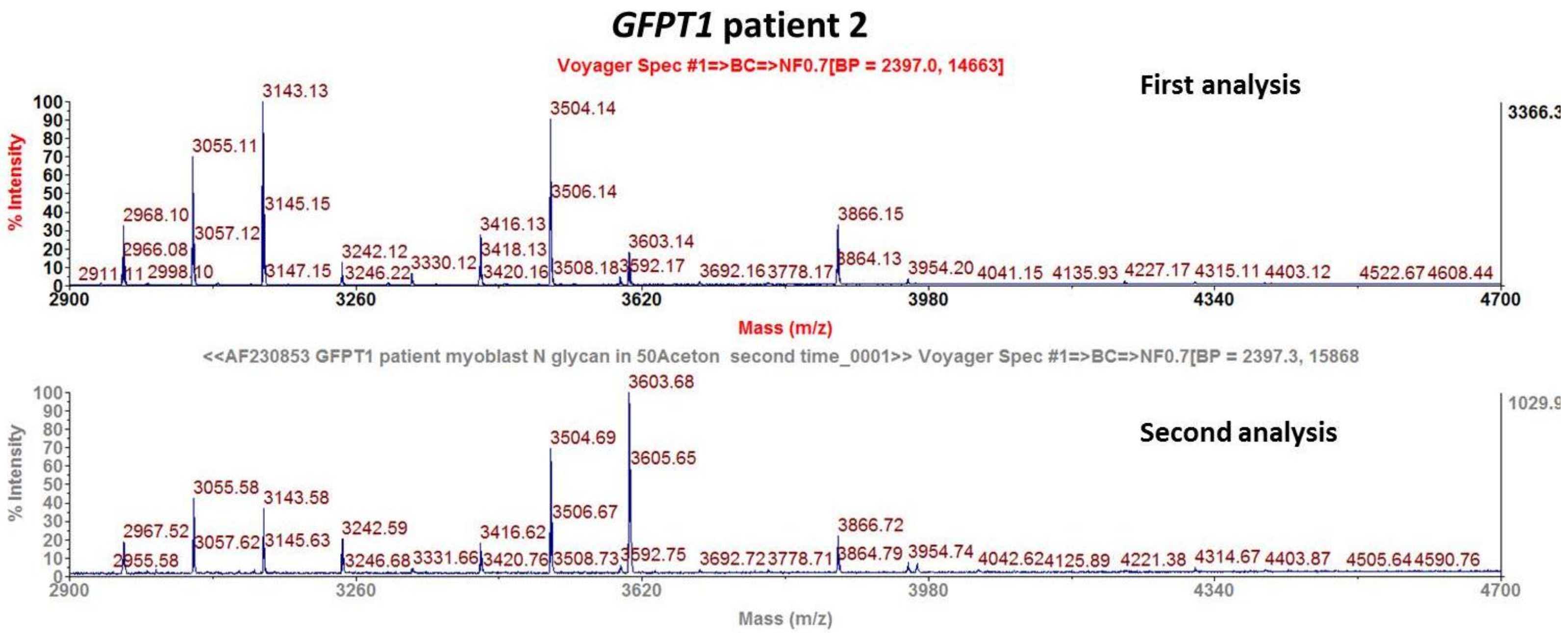

Anova: Single Factor						
SUMMARY						
Groups	Count	Sum	Average	Variance		
GFPT1 patient 2, first analysis	14	3.7134	0.2652	0.1245		
GFPT1 patient 2, second analysis	14	2.4057	0.1718	0.0396		
ANOVA						
Source of Variation	SS	df	MS	F	P-value	F critical
Between Groups	0.06108	1	0.0611	0.7445	0.3961	4.2252
Within Groups	2.13296	26	0.082			
Total	2.19404	27				

(**F**)

Figure S5. *Cont.*

***DOK7* patient**

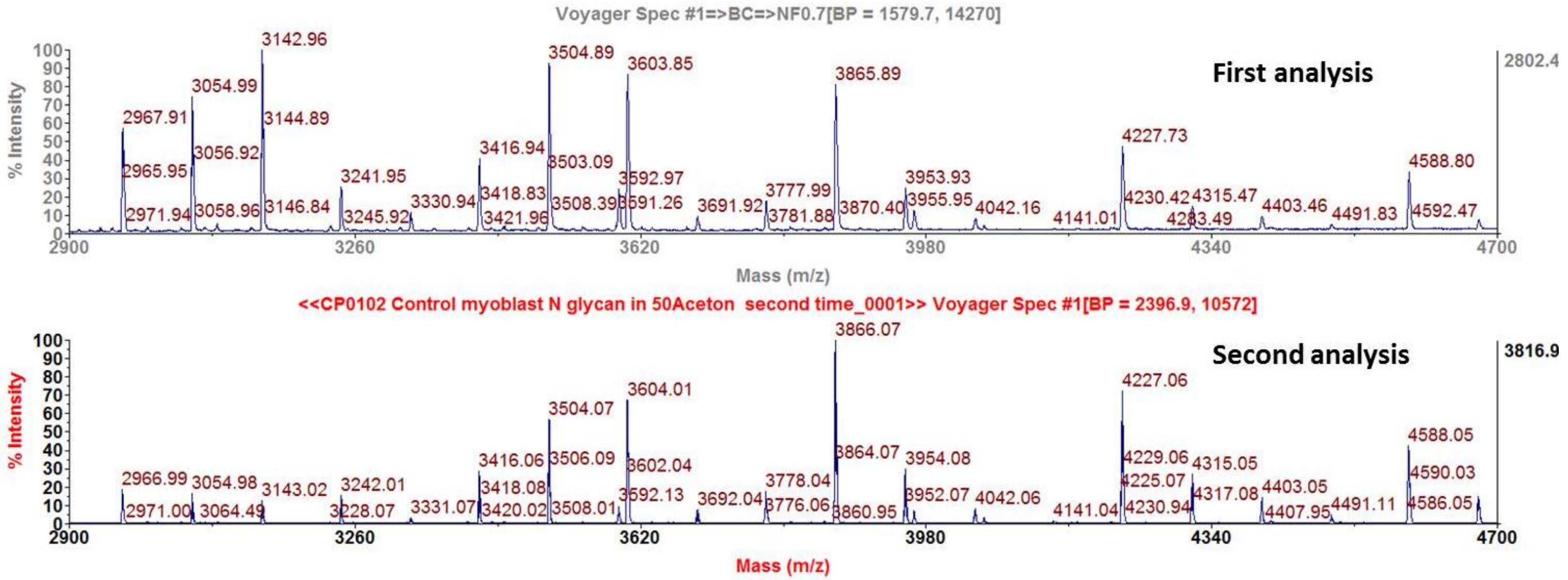

Anova: Single Factor						
SUMMARY						
Groups	Count	Sum	Average	Variance		
DOK7 patient, first analysis	14	6.2575	0.447	0.0991		
DOK7 patient, second analysis	14	4.5356	0.324	0.0722		
ANOVA						
Source of Variation	SS	df	MS	F	P-value	F critical
Between Groups	0.1059	1	0.1059	1.236	0.2764	4.2252
Within Groups	2.22756	26	0.0857			
Total	2.33346	27				

(**G**)

Figure S5. *Cont.*

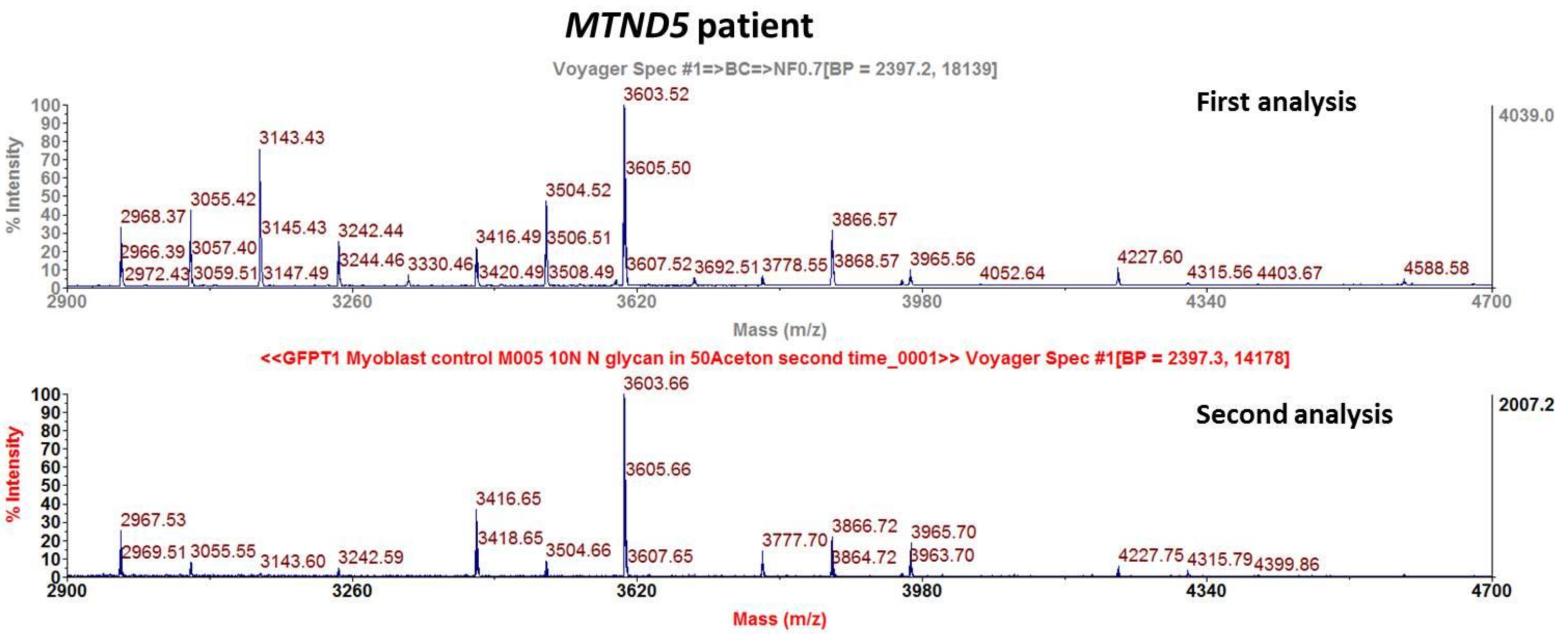

Anova: Single Factor						
SUMMARY						
Groups	Count	Sum	Average	Variance		
MTND5 patient, first analysis	14	2.892	0.2066	0.0505		
MTND5 patient, second analysis	14	1.3366	0.0955	0.0121		
ANOVA						
Source of Variation	SS	df	MS	F	P-value	F critical
Between Groups	0.0864	1	0.0864	2.759	0.1087	4.2252
Within Groups	0.81422	26	0.0313			
Total	0.90063	27				

(**H**)

Figure S5. *Cont.*

Additional Samples

Anova: Single Factor						
SUMMARY						
Groups	Count	Sum	Average	Variance		
myoblast healthy control 1	14	5.1907	0.3708	0.1193		
myoblast healthy control 2	14	3.7575	0.2684	0.0586		
GFPT1 patient 1	14	5.4019	0.3858	0.1034		
GFPT1 patient 2	14	3.7134	0.2652	0.1245		
DOK7 patient	14	6.2575	0.447	0.0991		
MTND5 patient	14	2.892	0.2066	0.0505		
LGMD2A patient	14	4.5738	0.3267	0.0855		
Pompe disease patient	14	5.9821	0.4273	0.1642		
ANOVA						
Source of Variation	SS	df	MS	F	P-value	F critical
Between Groups	0.71043	7	0.1015	1.0085	0.4297	2.0989
Within Groups	10.4658	104	0.1006			
Total	11.1763	111				

(I)

Figure S5. *Cont.*

ANOVA analysis of MS/MS data

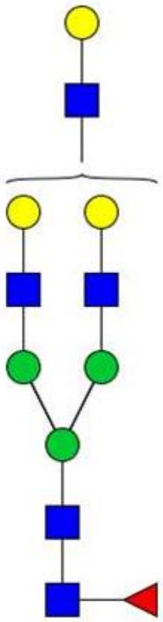
m/z 2693

Anova: Single Factor							
SUMMARY							
Groups	Count	Sum	Average	Variance			
myoblast healthy control 1	7	1.846981	0.263854	0.110998			
myoblast healthy control 2	7	1.744782	0.249255	0.117039			
GFPT1 patient 1	7	1.773967	0.253424	0.114121			
GFPT1 patient 2	7	1.658956	0.236994	0.118663			
DOK7 patient	7	1.497809	0.213973	0.122863			
MTND5 patient	7	1.638627	0.23409	0.116751			
LGMD2A patient	7	1.424044	0.203435	0.12499			
Pompe disease patient	7	1.613139	0.230448	0.118103			
ANOVA							
Source of Variation	SS	df	MS	F	P-value	F critical	
Between Groups	0.019848	7	0.002835	0.024042	0.999984	2.207436	
Within Groups	5.661162	48	0.117941				
Total	5.681011	55					

(**J**)

Figure S5. *Cont.*

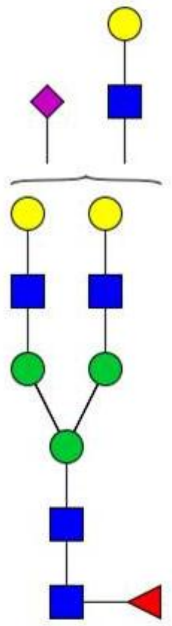

m/z 3055

Anova: Single Factor						
SUMMARY						
Groups	Count	Sum	Average	Variance		
myoblast healthy control 1	15	3.221139	0.214743	0.084465		
myoblast healthy control 2	15	3.007335	0.200489	0.077004		
GFPT1 patient 1	15	3.217751	0.214517	0.077672		
GFPT1 patient 2	15	3.143243	0.20955	0.067485		
DOK7 patient	15	2.827649	0.18851	0.072329		
MTND5 patient	15	2.869131	0.191275	0.072697		
LGMD2A patient	15	2.887922	0.192528	0.07004		
Pompe disease patient	15	3.211432	0.214095	0.073406		
ANOVA						
Source of Variation	SS	df	MS	F	P-value	F critical
Between Groups	0.013493	7	0.001928	0.025913	0.99998<br>1	2.092381
Within Groups	8.331362	112	0.074387			
Total	8.344855	119				

(**K**)

Figure S5. *Cont.*

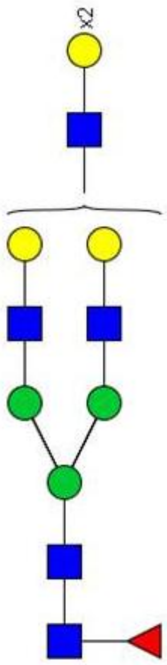

Anova: Single Factor						
SUMMARY						
Groups	Count	Sum	Average	Variance		
myoblast healthy control 1	10	1.554012	0.155401	0.090348		
myoblast healthy control 2	10	1.605102	0.16051	0.088959		
GFPT1 patient 1	10	1.543141	0.154314	0.09061		
GFPT1 patient 2	10	1.602429	0.160243	0.090208		
DOK7 patient	10	1.529101	0.15291	0.09086		
MTND5 patient	10	1.531205	0.15312	0.090842		
LGMD2A patient	10	1.395141	0.139514	0.092785		
Pompe disease patient	10	1.498925	0.149892	0.091328		
ANOVA						
Source of Variation	SS	df	MS	F	P-value	F critical
Between Groups	0.003075	7	0.000439	0.00484	1	2.139656
Within Groups	6.533456	72	0.090742			
Total	6.536531	79				

(**L**)

Figure S5. *Cont.*

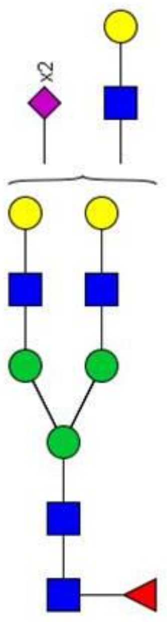

m/z 3416

Anova: Single Factor						
SUMMARY						
Groups	Count	Sum	Average	Variance		
myoblast healthy control 1	12	3.666897	0.305575	0.187442		
myoblast healthy control 2	12	3.41037	0.284198	0.102746		
GFPT1 patient 1	12	3.039815	0.253318	0.094479		
GFPT1 patient 2	12	3.2147	0.267892	0.102098		
DOK7 patient	12	3.533928	0.294494	0.151509		
MTND5 patient	12	3.290426	0.274202	0.147745		
LGMD2A patient	12	3.415446	0.284621	0.146992		
Pompe disease patient	12	3.739437	0.31162	0.201239		
ANOVA						
Source of Variation	SS	df	MS	F	P-value	F critical
Between Groups	0.031606	7	0.004515	0.031846	0.99996	2.115472
Within Groups	12.47676	88	0.141781			
Total	12.50837	95				

(**M**)

Figure S5. *Cont.*

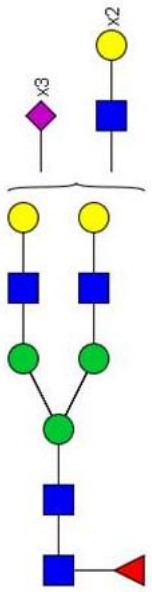

m/z 4226

Anova: Single Factor						
SUMMARY						
Groups	Count	Sum	Average	Variance		
myoblast healthy control 1	12	3.765016	0.313751	0.166213		
myoblast healthy control 2	12	2.44387	0.203656	0.084358		
GFPT1 patient 1	12	3.092784	0.257732	0.093789		
GFPT1 patient 2	12	3.830986	0.319249	0.09056		
DOK7 patient	12	2.797297	0.233108	0.105456		
MTND5 patient	12	2.521792	0.210149	0.102864		
LGMD2A patient	12	2.97479	0.247899	0.115249		
Pompe disease patient	12	3.843511	0.320293	0.329943		
ANOVA						
Source of Variation	SS	df	MS	F	P-value	F critical
Between Groups	0.197831	7	0.028262	0.207723	0.982901	2.115472
Within Groups	11.97276	88	0.136054			
Total	12.17059	95				

(N)

Figure S5. ANOVA analysis of myoblasts MS data (**A**–**I**) and MS/MS data (**J**–**N**). ■ GlcNAc, ● Man, ○ Gal, ▲ Fuc, ◆ NeuAc.

© 2015 by the authors; licensee MDPI, Basel, Switzerland. This article is an open access article distributed under the terms and conditions of the Creative Commons Attribution license (http://creativecommons.org/licenses/by/4.0/).
